# The Signal Transduction Protein P_II_ Controls Ammonium, Nitrate and Urea Uptake in Cyanobacteria

**DOI:** 10.3389/fmicb.2019.01428

**Published:** 2019-06-25

**Authors:** Björn Watzer, Philipp Spät, Niels Neumann, Moritz Koch, Roman Sobotka, Boris Macek, Oliver Hennrich, Karl Forchhammer

**Affiliations:** ^1^Interfaculty Institute of Microbiology and Infection Medicine Tübingen, Department of Organismic Interactions, University of Tübingen, Tübingen, Germany; ^2^Interfaculty Institute for Cell Biology, Department of Quantitative Proteomics, University of Tübingen, Tübingen, Germany; ^3^Centre Algatech, Institute of Microbiology, Academy of Sciences of the Czech Republic, Třeboň, Czechia

**Keywords:** P_II_ signaling protein, GlnB, cyanobacteria, nitrogen regulation, nitrate uptake, ammonium uptake, urea uptake, ABC transporters

## Abstract

P_II_ signal transduction proteins are widely spread among all domains of life where they regulate a multitude of carbon and nitrogen metabolism related processes. Non-diazotrophic cyanobacteria can utilize a high variety of organic and inorganic nitrogen sources. In recent years, several physiological studies indicated an involvement of the cyanobacterial P_II_ protein in regulation of ammonium, nitrate/nitrite, and cyanate uptake. However, direct interaction of P_II_ has not been demonstrated so far. In this study, we used biochemical, molecular genetic and physiological approaches to demonstrate that P_II_ regulates all relevant nitrogen uptake systems in *Synechocystis* sp. strain PCC 6803: P_II_ controls ammonium uptake by interacting with the Amt1 ammonium permease, probably similar to the known regulation of *E. coli* ammonium permease AmtB by the P_II_ homolog GlnK. We could further clarify that P_II_ mediates the ammonium- and dark-induced inhibition of nitrate uptake by interacting with the NrtC and NrtD subunits of the nitrate/nitrite transporter NrtABCD. We further identified the ABC-type urea transporter UrtABCDE as novel P_II_ target. P_II_ interacts with the UrtE subunit without involving the standard interaction surface of P_II_ interactions. The deregulation of urea uptake in a P_II_ deletion mutant causes ammonium excretion when urea is provided as nitrogen source. Furthermore, the urea hydrolyzing urease enzyme complex appears to be coupled to urea uptake. Overall, this study underlines the great importance of the P_II_ signal transduction protein in the regulation of nitrogen utilization in cyanobacteria.

## Introduction

The emergence of the oxygenic photosynthesis by ancestors of present cyanobacteria (Soo et al., 2017) laid the ground for the evolution of present days life on planet earth. Until today, cyanobacteria occupy a high variety of illuminated habitats, where they represent one of the most abundant primary producers (Whitton, 2012). Accordingly, cyanobacteria are essential contributors to the global carbon cycle. Many cyanobacterial strains have acquired the ability to fix atmospheric nitrogen, making them key players in the global nitrogen turnover (Herrero and Flores, 2008). Nitrogen represents a necessary macronutrient for all living organisms and therefore constitutes an important growth-limiting factor in most ecosystems (Vitousek and Howarth, 1991). The regulation of nitrogen metabolism in cyanobacteria mainly depends on the fine-tuned network of the signal transduction protein P_II_, the global nitrogen transcription factor NtcA and the NtcA co-activator PipX (Vegapalas et al., 1992; Espinosa et al., 2006, 2007, 2014; Forchhammer, 2008; Luque and Forchhammer, 2008; Forcada-Nadal et al., 2018).

P_II_ signal-transduction proteins are widespread in all three domains of life, where they represent one of the largest and most ancient families of signaling proteins (Chellamuthu et al., 2013; Forchhammer and Luddecke, 2016). P_II_ proteins are involved in the regulation of various nitrogen- and carbon-anabolic processes (Forchhammer, 2004, 2008). Canonical P_II_ proteins are homo-trimeric with three characteristic loop regions, designated as B-, C-, and T-loops, which compose the effector molecule binding sites (Cheah et al., 1994; Xu et al., 2003; Forchhammer, 2004, 2008; Llacer et al., 2007; Fokina et al., 2010a; Zhao et al., 2010; Zeth et al., 2014). The large surface exposed T-loop is the prevailing protein interaction module of P_II_. The P_II_ proteins sense the energy status of the cell by the competitive binding of ADP or ATP (Zeth et al., 2014). Binding of ATP and synergistic binding of 2-oxoglutarate (2-OG) allows P_II_ to sense the current carbon/nitrogen status of the cell (Fokina et al., 2010a). 2-OG is an intermediate of the TCA-cycle that provides the carbon skeleton for inorganic nitrogen incorporation by the glutamine synthetase/glutamate synthase (GS/GOGAT) cycle. Due to this, 2-OG links carbon and nitrogen metabolism and acts as an indicator for the intracellular carbon/nitrogen balance (Muro-Pastor et al., 2001; Fokina et al., 2010a). Besides effector molecule binding, post-translational modification of P_II_ represents a second level of regulation (Forchhammer et al., 2004; Merrick, 2014). Depending on nitrogen availability, cyanobacterial P_II_ can be phosphorylated at the apex of the T-loop at position Ser49 (Forchhammer and Tandeau de Marsac, 1995; Forchhammer and Hedler, 1997). In other prokaryotes, like *E. coli*, P_II_ is modified by uridylylation in response to nitrogen availability instead of phosphorylation (Jiang et al., 1998). Binding of effector molecules as well as post-translational modifications lead to various P_II_ conformations. Depending on the conformational state, P_II_ can interact with a variety of interaction partners and thereby regulate the cellular C/N balance (Radchenko and Merrick, 2011; Forchhammer and Luddecke, 2016). In cyanobacteria, P_II_ indirectly regulates the global nitrogen control transcriptional factor NtcA through binding of the NtcA co-activator PipX (Espinosa et al., 2007). In common with other bacteria, the cyanobacterial P_II_ protein can control the acetyl-CoA levels by interacting with the biotin carboxyl carrier protein (BCCP) of acetyl-CoA carboxylase (ACC) (Hauf et al., 2016). Furthermore, P_II_ regulates arginine biosynthesis by interacting with the enzyme *N*-acetylglutamate kinase (NAGK), which catalyzes the rate-limiting step of this pathway (Caldovic and Tuchman, 2003; Heinrich et al., 2004; Llacer et al., 2007; Watzer et al., 2015). If sufficient energy and nitrogen is available, indicated by a high intracellular ATP and low 2-OG level, non-phosphorylated P_II_ interacts with NAGK, enhancing its catalytic efficiency and relieving it from feedback inhibition by arginine (Heinrich et al., 2004; Maheswaran et al., 2004; Llacer et al., 2007). At high intracellular arginine levels, the carbon/nitrogen storage polymer cyanophycin (multi-L-arginyl-poly-L-aspartate) accumulates in *Synechocystis* sp. strain PCC 6803 (hereafter *Synechocystis*) (Maheswaran et al., 2006; Watzer et al., 2015). A P_II_ variant was identified with a single amino acid substitution, Ile86 to Asn86 [thereafter referred as P_II_(I86N)], which constitutively binds NAGK *in vitro* (Fokina et al., 2010b). Replacing the wild-type P_II_ with a I86N variant in *Synechocystis* generated a mutant strain, which strongly overproduced arginine and cyanophycin (Watzer et al., 2015). On the other hand, the P_II_(I86N) strain showed a growth defect in ammonium-supplemented medium (Watzer et al., 2015).

Cyanobacteria use nitrogen sources in a hierarchical order, with ammonium being the preferred nitrogen source. As a consequence, when ammonium is provided together with other suitable nitrogen sources, ammonium will be utilized first (Muro-Pastor et al., 2005). In most natural habitats, the ammonium availability is low, so that high affinity ammonium permeases are required for efficient ammonium uptake (Rees et al., 2006). In *Synechocystis*, the Amt1 permease is mainly responsible for ammonium uptake (Montesinos et al., 1998). However, elevated intracellular ammonium concentrations are toxic to the cells (Drath et al., 2008; Dai et al., 2014), and therefore, ammonium transport must be tightly controlled. The ammonium transporter family (Amt) is widespread among all domains of life (Wirén and Merrick, 2004). In *E. coli*, the P_II_ homolog GlnK regulates the ammonium permease AmtB by direct protein–protein interaction. Under ammonium excess conditions, ADP-complexed GlnK blocks an uncontrolled influx of ammonium by inserting the apex of the T-loop into the cytoplasmic exit pores of AmtB (Conroy et al., 2007).

For the assimilation of nitrate, an active nitrate transporter, a nitrate reductase (NR) and a nitrite reductase (NiR) are required (Ohashi et al., 2011). Two types of nitrate transporter systems have been found among cyanobacteria, a high-affinity nitrate/nitrite permease NrtP and the ABC-type transporter NrtABCD (NRT) (Omata et al., 1993; Luque et al., 1994; Sakamoto et al., 1999; Ohashi et al., 2011). NRT is a bispecific nitrate/nitrite transporter showing high affinity for both substrates (Maeda and Omata, 1997). Intracellular nitrate is first reduced to nitrite by NR and subsequently reduced to ammonium by NiR. Subsequently, ammonium is assimilated in the GS/GOGAT cycle (Flores and Herrero, 1994). Both, NR and NiR, use photosystem I reduced ferredoxin as an electron donor, indicating a coupling of photosynthesis and nitrate assimilation (Manzano et al., 1976; Flores and Herrero, 1994). Addition of ammonium to nitrate adapted cells results in an immediate inhibition of nitrate uptake and a repression of proteins involved in nitrate assimilation (NR and NiR). The ammonium-induced inhibition of NRT is regulated by the P_II_ protein and the C-terminal domain of NrtC (Kobayashi et al., 1997; Lee et al., 1998). Phosphomimetic variants of P_II_ and a P_II_ phosphatase (PphA) deletion mutant, in which P_II_ is constitutively phosphorylated, showed ammonium promoted inhibition of nitrate uptake like the wild-type (Lee et al., 2000; Kloft and Forchhammer, 2005). However, this response was abolished in a P_II_ deficient mutant (Kloft and Forchhammer, 2005).

The ability to utilize urea as nitrogen source is widely distributed among bacteria, fungi and algae (Baker et al., 2009; Solomon et al., 2010; Esteves-Ferreira et al., 2018). In common with other bacteria, the cyanobacteria *Synechocystis* and *Anabaena* sp. PCC 7120 possess a high affinity urea ABC-type transporter, which is capable of urea import at concentrations lower than 1 μM (Valladares et al., 2002). The gene cluster *urtABCDE*, encoding all subunits of this ABC-type urea transporter, is transcriptionally controlled by the global transcription factor NtcA (Valladares et al., 2002).

The present study was inspired by the phenotype of the cyanophycin-accumulating strain variant *Synechocystis* P_II_(I86N), which was impaired in ammonium utilization. Starting with analyzing a possible regulation of the cyanobacterial Amt1 permease by P_II_, we found additional evidence for a direct regulation of the nitrate/nitrite transporter NrtABCD and the urea transporter UrtABCDE by the P_II_ signaling protein during this study.

## Materials and Methods

### Cultivation Conditions

Standard cloning procedures were performed in *Escherichia coli* NEB 10-beta (NEB). Strains were grown in LB-medium at 37°C with constant shaking at 300 rpm.

*Synechocystis* strains were grown photoautotrophically in BG-11 medium supplemented with 5 mM NaHCO_3_ and nitrate, ammonium, or urea as nitrogen source (Rippka et al., 1979). BG-11 agar plates were produced by adding 1.5% (w/v) Bacto-agar (Difco), 0.3% (w/v) sodium thiosulfate pentahydrate and 10 mM TES-NaOH pH 8 (Roth) to liquid BG-11 medium. Antibiotics were added when required. Cultivation of liquid cultures for physiological experiments occurred in 50, 100, or 500 mL Erlenmeyer flasks, at 28°C and with constant shaking of 120 rpm. Cultures were continuously illuminated with a photon flux rate of 40–50 μE. Growth rates were determined by measuring the optical density at 750 nm (OD_750_).

For induction of nitrogen starvation conditions, exponentially growing cells (OD_750_ 0.4–0.8) were harvested by centrifugation (3,000 × *g* for 10 min at room temperature), washed and resuspended in BG-11 medium lacking a suitable nitrogen source (BG-11^0^).

For spot assays (drop plate method), *Synechocystis* cultures were adjusted to an OD_750_ of 1. A dilution series to the power of 10 was made using BG-11^0^. Then, 5 μL of every dilution step (10^0^–10^-4^) were dropped on BG-11 agar plates. Plates were cultivated at 28°C with constant illumination of 40 μmol of photons s^-1^ m^-2^.

### Bacterial Two-Hybrid Assay

Plasmids were constructed by PCR amplification using high-fidelity Q5 polymerase (NEB) and oligonucleotides with overlapping regions. Genomic *Synechocystis* DNA or plasmids served as templates. PCR fragments were inserted in linearized bacterial two-hybrid vectors pUT18 and pKT25 containing the genes for either the T18 or T25 subunit of the adenylate cyclase CyaA (Karimova et al., 2001) by isothermal, single-reaction DNA assembly according to Gibson et al. (2009). Since the multiple cloning site of pKT25 is located downstream of the T25 subunit, allowing only N-terminal localization of the tag, we constructed plasmid pKT25n (Table 1; pKT25n_fw and pKT25n_rev) to achieve a C-terminal fusion of the tag to the gene of interest. Therefore, plasmid pKT25 was linearized using PCR and the gene of interest was fused upstream of the T25 subunit. Primers, plasmids, and strains used in this study are listed in Tables 1–3, respectively.

**Table 1:** Oligonucleotides used in this study.

Primer	Sequence (5′–3′ direction)
pKT25n_fw	ACCATGCAGCAATCGCATCAG
pKT25n_rev	CATAGCTGTTTCCTGTGTGAAATTG
glnB_fw	TGTGTGGAATTGTGAGCGGATAACAATTTCACACAGGAAACAGCTATGAAAAAAGTAGAAGCGATTATTC
glnB_rev	CTCGCTGGCGGCTGAATTCGAGCTCGGTACCCGGGGATCAATAGCTTCGGTATCCTTTTC
pipX_fw	TTCACACAGGAAACAGCTATGAGTAACGAAATTTACCTTAAC
pipX_rev	GATGCGATTGCTGCATGGTAAAAGTGTTTTTATGTAACTTTG
pipX_pKT25n_fw	AAGTTACATAAAAACACTTTTACCATGCAGCAATCGCATCAG
pipX_pKT25n_rev	TAAGGTAAATTTCGTTACTCATAGCTGTTTCCTGTGTGAAATTG
amt1_pKT25_fw	CTGGCGCGCACGCGGCGGGCTGCAGGGTCGACTCTAGAGATGTCTAATTCGATATTGTCTAAAC
amt1_pKT25_rev	AAAACGACGGCCGAATTCTTAGTTACTTAGGTACCCGGGGATCTTATTCAGGGACAGTGG
amt1_fw	AACAATTTCACACAGGAAACAGCTATGTCTAATTCGATATTGTCTAAAC
amt1_rev	TGATGCGATTGCTGCATGGTTTCAGGGACAGTGGCACCG
amt1_pKT25n_fw	TCTCCGGTGCCACTGTCCCTGAAACCATGCAGCAATCGCATC
amt1_pKT25n_rev	ACAATATCGAATTAGACATAGCTGTTTCCTGTGTGAAATTGTTATCCGC
nrtC_pKT25_fw	CGCGCACGCGGCGGGCTGCAGGGTCGACTCTAGAGGATCCCCCCTTCATTGAAATTGATCATGTTG
nrtC_pKT25_rev	AGTCACGACGTTGTAAAACGACGGCCGAATTCTTAGTTATTGATTAACTTGATCAATTTGGTCGATGAG
nrtC_fw	AATTTCACACAGGAAACAGCTATGCCCTTCATTGAAATTGATCATG
nrtC_rev	CTGATGCGATTGCTGCATGGTTTGATTAACTTGATCAATTTGG
nrtC_pKT25n_fw	AAATTGATCAAGTTAATCAAACCATGCAGCAATCGCATCAG
nrtC_pKT25n_rev	TCAATTTCAATGAAGGGCATAGCTGTTTCCTGTGTGAAATTG
nrtD_pKT25_fw	CGCACGCGGCGGGCTGCAGGGTCGACTCTAGAGGATCCCCAAACAATGAATGTCAATGACCCTATCC
ntrD_pKT25_rev	CCCAGTCACGACGTTGTAAAACGACGGCCGAATTCTTAGTTAAGACCCTTCCATGGATTCCACTGAGGGGGTAG
nrtD_fw	AATTTCACACAGGAAACAGCTATGCAAACAATGAATGTCAATGACCCTATC
nrtD_rev	CTGATGCGATTGCTGCATGGTAGACCCTTCCATGGATTCCACTGAG
nrtD_pKT25n_fw	TGGAATCCATGGAAGGGTCTACCATGCAGCAATCGCATCAG
nrtD_pKT25n_rev	ATTGACATTCATTGTTTGCATAGCTGTTTCCTGTGTGAAATTG
urtD_pKT25_fw	CGCACGCGGCGGGCTGCAGGGTCGACTCTAGAGGATCCCACCAGCAAAATCTTAGAAATTCAAG
urtD_pKT25_rev	CCCAGTCACGACGTTGTAAAACGACGGCCGAATTCTTAGCTAATCTCCATCCTCATCAAC
urtD_fw	ACACAGGAAACAGCTATGACCAGCAAAATCTTAGAAATTCAAGAC
urtD_rev	GATGCGATTGCTGCATGGTATCTCCATCCTCATCAACACTG
urtD_pKT25n_fw	AGTGTTGATGAGGATGGAGATACCATGCAGCAATCGCATCAG
urtD_pKT25n_rev	TTCTAAGATTTTGCTGGTCATAGCTGTTTCCTGTGTGAAATTG
urtE_fw	GCGCGCACGCGGCGGGCTGCAGGGTCGACTCTAGAGGATGCTATGTTATCCTTTCCCCCATTCTTG
urtE_rev	CCCAGTCACGACGTTGTAAAACGACGGCCGAATTCTTAGTTATACTGCCAAAAATTTTTGGATAAC
urtE_fw	ACAATTTCACACAGGAAACAGCTATGGCTATGTTATCCTTTCCC
urtE_rev	TGATGCGATTGCTGCATGGTTACTGCCAAAAATTTTTGGATAACC
urtE_pKT25n_fw	TATCCAAAAATTTTTGGCAGTAACCATGCAGCAATCGCATCAG
urtE_pKT25n_rev	GGGAAAGGATAACATAGCCATAGCTGTTTCCTGTGTGAAATTG

**Table 2:** Plasmids used in this study.

Plasmid	Tag localization	Description	References
pPD-*N*FLAG	N-terminal	Encoding the N-terminal 3xFLAG tag	Hollingshead et al., 2012
pPD-*C*FLAG	C-terminal	Encoding the C-terminal 3xFLAG tag	Chidgey et al., 2014
pKT25		Encoding T25 fragment of adenylate cyclase CyaA (amino acids 1–224)	Karimova et al., 2001
pKT25n		Derived from pKT25. Upstream of the T25 fragment	This study
pUT18		Encoding T18 fragment of adenylate cyclase CyaA (amino acids 225–399)	Karimova et al., 2001
pUT18 *gln*B	N-terminal	Derived from pUT18. Encoding *gln*B	This study
pUT18 *gln*B (I86N)	N-terminal	Derived from pUT18. Encoding *gln*B containing the I86N mutation	This study
pKT25n *pip*X	C-terminal	Derived from pKT25. Sequence encoding *pip*X. Positive control	This study
pKT25 *pip*X	N-terminal	Derived from pKT25. Sequence encoding *pip*X. Positive control	This study
pKT25n *amt*1	C-terminal	Derived from pKT25. Sequence encoding *amt*1	This study
pKT25 *amt*1	N-terminal	Derived from pKT25. Sequence encoding *amt*1	This study
pKT25n *nrt*C	C-terminal	Derived from pKT25. Sequence encoding *nrt*C	This study
pKT25 *nrt*C	N-terminal	Derived from pKT25. Sequence encoding *nrt*C	This study
pKT25n *nrt*D	C-terminal	Derived from pKT25. Sequence encoding *nrt*D	This study
pKT25 *nrt*D	N-terminal	Derived from pKT25. Sequence encoding *nrt*D	This study
pKT25n *urt*D	C-terminal	Derived from pKT25. Sequence encoding *urt*D	This study
pKT25 *urt*D	N-terminal	Derived from pKT25. Sequence encoding *urt*D	This study
pKT25n *urt*E	C-terminal	Derived from pKT25. Sequence encoding *urt*E	This study
pKT25 *urt*E	N-terminal	Derived from pKT25. Sequence encoding *urt*E	This study

**Table 3:** Strains used in this study.

Strains	Description	References
*E. coli* NEB 10-beta	Cloning strain	NEB
*E. coli* BTH101	Bacterial two-hybrid host strain	Euromedex
*Synechocystis* sp. PCC 6803	Wild type	Pasteur Culture Collection
*Synechocystis* sp. P_II_(I86N)	Genomic P_II_(I86N) mutant	Watzer et al., 2015
*Synechocystis* sp. ΔP_II_	Chromosomal deletion of *glnB*	Hisbergues et al., 1999
*Synechocystis* sp. ΔP_II_ + P_II_-Venus	*Synechocystis* sp. ΔP_II_ transformed with pVZ322 encoding a P_II_-Venus fusion	Hauf et al., 2016

*Escherichia coli* BTH101 cells were co-transformed with plasmid pUT18 and plasmid pKT25 or pKT25n (Table 2). Plasmids pKT25 or pKT25n contained the possible P_II_ interaction partner fused N- or C-terminal to the T25 subunit. Plasmid pUT18 contains a gene-fusion of the P_II_-encoding *gln*B gene or a genetically modified *gln*B gene containing the I86N mutation [Ile (5′ATC) at codon position 86 to Asn (5′AAC)] (Watzer et al., 2015), the R9L mutation [Arg (5′CGC) at codon position 9 to Leu (5′CTG)] (Fokina et al., 2010a) or the S49D mutation [Ser (5′TCG) at codon position 49 to Asp (5′GAT)] (Lee et al., 2000) with the T18 subunit. The *gln*B gene and the modified *gln*B genes were always fused N-terminal with the T18 subunit. Co-transformants were plated on LB-plates (supplemented with 100 μg mL^-1^ ampicillin and 50 μg mL^-1^ kanamycin) and cultivated for 2 days at 30°C.

To reduce the level of heterogeneity, five clones from each plate were picked to inoculate 5 mL LB-medium (containing 100 μg mL^-1^ ampicillin and 50 μg mL^-1^ kanamycin). Cultures were cultivated overnight at 37°C. Overnight cultures were diluted 1:100 in 3 mL of fresh LB-medium (containing 100 μg mL^-1^ ampicillin and 50 μg mL^-1^ kanamycin) and grown to an OD_600_ of 0.7. Three μL of each culture were plated on X-Gal (containing 100 μg mL^-1^ ampicillin, 50 μg mL^-1^ kanamycin, 1 mM IPTG, 40 μg mL^-1^ X-Gal) and MacConkey (containing 100 μg mL^-1^ ampicillin, 50 μg mL^-1^ kanamycin, 1 mM IPTG, 1% maltose) reporter plates. Reporter plates were incubated for 3–4 days at 25°C.

### Construction and Cultivation of *Synechocystis* P_II_-3xFLAG Tag Strains

The previously described pPD-*N*FLAG and pPD-*C*FLAG plasmids were used to construct *Synechocystis* P_II_ (GlnB) fusion proteins with an N- or C-terminal 3xFLAG tag, respectively (Hollingshead et al., 2012; Chidgey et al., 2014). Together with a kanamycin resistance cassette, this construct was inserted in the *Synechocystis* wild-type genome by homologous recombination, replacing the *psbAII* gene (Chidgey et al., 2014). Transformants were selected and segregated by kanamycin resistance. For pull-down experiments, 2 L batch cultures were inoculated at OD_750_ = 0.2 in BG-11 medium and propagated as described above, with magnetic stirring at 120 rpm and bubbling with 2% CO_2_ (v/v) supplemented ambient air. Cell harvesting was performed at OD_750_ = 0.6 by mixing the cultures with ice in a 2:1 ratio (w/w) for rapid metabolic inactivation, and centrifugation at 7,477 × *g* for 10 min. Cell pellets were subsequently washed with nitrogen-free BG-11^0^ at 4°C and snap frozen in liquid nitrogen. For experimental controls, the wild-type strain was similarly cultivated and subjected to pull-down assays followed by mass spectrometry analyses as described below. Two independent replicates were prepared per condition.

### Preparation of Cell Extracts and Anti-FLAG Pull-Down

Frozen cell pellets were washed with 5 mL IP buffer, containing 25 mM MES/NaOH; pH 6.5, 5 mM CaCl_2_, 10 mM MgCl_2_, and 20% (v/v) glycerol and subsequently resuspended in 3 mL IP buffer, including protease inhibitors (complete EDTA-free; Roche) for cell lysis. Therefore, an equal volume of glass beads (0.1–0.15 mm diameter) was added and cells were disrupted using a FastPrep-24 Ribolyser (MP Biomedicals) with five cycles of 20 s at 6.5 m s^-1^ and 4°C. After centrifugation for 5 min at 3,314 × *g* and 4°C, the supernatant was transferred and adjusted with IP buffer to a final volume of 6 mL. For membrane protein solubilization, 1.5% (w/v) dodecyl-β-D-maltoside (DDM; Carl Roth) was added and incubated under agitation for 1 h at 4°C, before insoluble cell debris was removed by centrifugation for 20 min at 20,000 × *g* and 4°C. The soluble cell extracts (from 2 L cultures) were subjected to the P_II_-FLAG pull-down procedure: for this, 400 μL of resuspended anti-FLAG-M2-agarose (Sigma) was added into empty SPE-columns and washed twice with each 1 mL IP buffer, supplemented with 0.04% (w/v) DDM, before the supernatants were incubated for 5 min and removed by gravity flow. Repeated washing steps with each 5 mL DDM supplemented IP buffer were performed similarly, until the flow through appeared colorless. Elution of coupled proteins from the anti-FLAG resin was performed by incubation in 600 μL DDM supplemented IP buffer containing 0.3 μg/mL 3xFLAG^TM^ peptide (Sigma-Aldrich) for 40 min.

### Proteomics Workflow, NanoLC-MS/MS Analysis, and Data Processing

Eluates from the pull-down workflow were subjected to acetone/methanol precipitation and resulting protein pellets were resuspended in denaturation buffer for subsequent tryptic digestion as described elsewhere (Spät et al., 2015). Resulting peptide mixtures were subjected to stage tip purification (Rappsilber et al., 2007). Protein detection by mass spectrometry (MS) was performed as described previously (Spät et al., 2015): in brief, peptide mixtures were loaded onto a 15 cm reversed-phase C_18_ nanoHPLC column on an EASY-LC system (Proxeon Biosystems) and separated in a 90 min segmented linear gradient. Eluted peptides were ionized by the on-line coupled ESI source and injected in a LTQ Orbitrap XL mass spectrometer (Thermo Scientific). MS spectra were acquired in the positive-ion mode. Per scan cycle, one initial full (MS) scan was followed by fragmentation of the 15 most intense multiply charged ions by collision induced dissociation (CID) for MS/MS scans. The scan range was 300–2,000 m/z for precursor ions at resolution 60,000 and sequenced precursors were dynamically excluded for fragmentation for 90 s. The lock mass option was enabled for real time mass recalibration (Olsen et al., 2005). All raw spectra were processed with the MaxQuant software (version 1.5.2.8) (Cox and Mann, 2008) at default settings. Peak lists were searched against a target-decoy database of *Synechocystis* sp. PCC 6803 with 3,671 protein sequences, retrieved from Cyanobase (Nakao et al., 2010) (July 2014), plus the sequence of the N- or C-terminal 3xFLAG tagged P_II_ fusion protein (MDYKDDDDKDYKDDDDKDYKDDDDKAAAKKVEAIIRPF KLDEVKIALVNAGIVGMTVSEVRGFGRQKGQTERYRGSEYT VEFLQKLKIEIVVDEGQVDMVVDKLVSAARTGEIGDGKIFIS PVDSVVRIRTGEKDTEAI or MKKVEAIIRPFKLDEVKIALVN AGIVGMTVSEVRGFGRQKGQTERYRGSEYTVEFLQKLKIEIV VDEGQVDMVVDKLVSAARTGEIGDGKIFISPVDSVVRIRTG EKDTEAIASYKDDDDKDYKDDDDKDYKDDDDK), respectively, as well as 245 common contaminants with the following database search criteria: trypsin was defined as a cleaving enzyme and up to two missed cleavages were allowed; carbamido-methylation of cysteines was defined as a fixed modification and methionine oxidation and protein N-terminal acetylation as variable modifications. Peptide and protein false discovery rates retrieved from MaxQuant were limited to 1%, each. Raw data acquired by mass spectrometry was deposited at the ProteomeXchange Consortium via the Pride partner repository (Vizcaino et al., 2013) under the identifier PXD013411.

### Determination of Nitrate, Nitrite, Ammonium, and Urea in Cell-Free Culture Medium

To determine the nitrate, ammonium, or urea uptake, exponentially growing cells (OD_750_ = 0.4–0.8) were harvested by centrifugation (3,000 × *g* for 10 min at room temperature) and washed twice with BG-11^0^ medium lacking combined nitrogen sources. Subsequently, the cultures were adjusted to OD_750_ = 1 with BG-11^0^ medium. The assays were started by adding either 200 μM NaNO_3_, 200 μM NH_4_^+^ or 150 μM urea, respectively. Cells were cultivated under constant shaking of 120 rpm and illumination of 40–50 μE. 1 mL aliquots of the cell suspensions were taken and subsequently centrifuged (13,000 × *g* for 5 min at room temperature) to remove the cells.

For nitrate quantification, the absorbance at 210 nm was measured in the cell-free medium. Since both nitrate and nitrite absorb at 210 nm, the apparent nitrate values were corrected for the presence of nitrite (Kloft and Forchhammer, 2005). Nitrite concentration of the cell-free medium was determined using the modified Griess reaction according to Fiddler (1977).

The ammonium concentration of cell-free medium was measured by using the Nessler reaction (Vogel et al., 1989). Urea was quantified by using the urea nitrogen (BUN) colorimetric detection kit (Invitrogen).

### Microscopy Procedures and Image Evaluation

Fluorescence microscopy was performed using a Leica DM5500B microscope with a 100×/1.3 oil objective. For the detection of Venus fluorescence, an ET500/20× excitation filter and an ET535/30m emission filter were used referred as YFP-channel. To detect cyanobacterial autofluorescence, an excitation filter BP 535/50 and a suppression filter BP 610/75 were used. Image acquisition was done with a Leica DFC360FX black-and-white camera. Captured images were colored with the Leica Application Suite Software (LAS AF) provided by Leica Microsystems. Bright-field images were exposed for 6 ms, Venus fluorescence images for 150 ms and autofluorescence images for 100 ms. Microscope slides covered with dried 2% (w/v) agarose solution were used to immobilize the cells during all microscopical examinations.

Quantitative image evaluation by fluorescence intensity measurements was performed using the open-source software ImageJ (Fiji) (Schindelin et al., 2012). To estimate the fluorescence intensities in different cell compartments, a linear profile of the gray values across the cells through the plasma membrane and cytoplasm was determined. The average gray level of the cytoplasm was subtracted from the maximum gray level of the plasma membrane signal to yield the “Δ fluorescence” value, which indicates the cytoplasmic membrane-localized signal. An example of this profile data quantification is given in Supplementary Figure 1.

## Results

### P_II_ Signaling Mutants Show Increased Sensitivity to Ammonium

P_II_ homologs of the GlnK subfamily from heterotrophic bacteria are known to regulate cellular ammonium influx by direct protein–protein interaction with the ammonium permease AmtB (Huergo et al., 2012). The observation of impaired ammonium utilization in the *Synechocystis* strain, which harbors the P_II_(I86N) variant, suggested that this P_II_ variant either blocked ammonium uptake or abolished proper ammonium uptake regulation with consequent ammonium sensitivity. The Ile 86 to Asn point mutation in this P_II_ variant causes a specific alteration of the T-loop conformation, which facilitates interaction with NAGK and thus, results in an over-activation of this P_II_ target (Fokina et al., 2010b). Conversely, other P_II_ targets might by negatively affected by the altered T-loop conformation. To gain further insights into the role of P_II_ in ammonium uptake, we tested ammonium utilization of a P_II_ deletion mutant (ΔP_II_) (Hisbergues et al., 1999) and of the ΔP_II_ strain complemented with P_II_-Venus (P_II_-Venus-comp.) (Hauf et al., 2016). The complementation strain was previously generated by introducing a shuttle vector (pVZ322), encoding a P_II_-Venus fusion under control of the native *gln*B promoter into the *Synechocystis* ΔP_II_ mutant (Hauf et al., 2016). The Venus fluorescent protein was fused to the C-terminus of P_II_ to avoid sterical hindrance of the T-loop.

**FIGURE 1:**
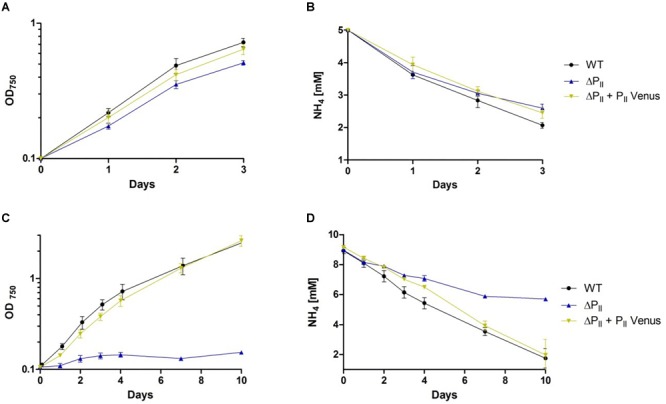
Ammonium supplemented growth and ammonium utilization of *Synechocystis* sp. wild-type, ΔP_II_ and P_II_-Venus-comp. (ΔP_II_ + P_II_-Venus) strains. Values are the means of three biological replicates. **(A)** Growth curve in presence of 5 mM ammonium. **(B)** Ammonium concentration in culture supernatants of the various *Synechocystis* strains grown in BG-11 medium with 5 mM ammonium. **(C)** Growth curve in presence of 10 mM ammonium. **(D)** Ammonium concentration in culture supernatants of the various *Synechocystis* strains grown in BG-11 medium with 10 mM ammonium.

**FIGURE 2:**
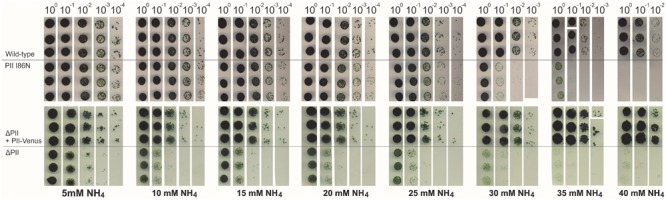
Ammonium sensitivity of the various *Synechocystis* strains (as in Figure 1) as determined by the drop plate method with three biological replicates per strain. Starved cultures were adjusted to an OD_750_ of 1.0 and diluted tenfold in series. The dilutions were dropped onto BG-11 agar plates containing increasing concentrations of ammonia (5–40 mM).

When grown in presence of 5 mM ammonium in liquid medium, the *Synechocystis* wild-type, the ΔP_II_ and the P_II_-Venus-comp. strains showed similar growth rates and ammonium consumption (Figure 1A,B). However, with an elevated ammonium concentration of 10 mM, only the wild-type and P_II_-Venus-comp. strains could maintain normal growth, while the ΔP_II_ mutant arrested growth (Figure 1C). Ammonium utilization of wild-type and P_II_-Venus-comp. were similar under this condition (Figure 1D) while the ΔP_II_ strain ceased ammonium utilization (Figure 1D). The ammonium-sensitive phenotype of the ΔP_II_ strain, in particular the response toward 10 mM ammonium, resembled the previously reported phenotype of the *Synechocystis* P_II_(I86N) strain (Watzer et al., 2015). One possibility to explain the inability of the P_II_ mutant strains to grow in 10 mM ammonium-supplemented medium is an increased sensitivity toward toxic effects of ammonium. Although ammonium represents the preferred nitrogen source, it becomes toxic for many photosynthetic organisms at higher concentrations (Drath et al., 2008). To find out whether the reduced growth and impaired ammonium utilization results from ammonium intoxication, we assayed the growth of the various strains in the presence of increasing concentrations of ammonium using the spot assay method (Figure 2). Over a range from 5 to 40 mM ammonium, the wild-type and P_II_-Venus-comp. strains showed a similar growth and ammonium tolerance. However, the P_II_(I86N) mutant showed impaired growth at 25 mM while the ΔP_II_ mutant was already strongly affected at a concentration of 10 mM ammonium (Figure 2). This demonstrated that P_II_ is indeed required to cope with elevated ammonium concentrations. The P_II_(I86N) mutant can only partially replace the wild-type P_II_ protein, whereas P_II_-Venus perfectly complemented the ΔP_II_ strain.

### Identification of Novel P_II_ Interaction Partners by FLAG-Tag P_II_ Pull-Down Assays

Uncontrolled influx of ammonium and resulting intoxication of *Synechocystis* ΔP_II_ and P_II_(I86N) mutants is likely a cause for the elevated ammonium sensitivity in these strains. This suggested the involvement of P_II_ in the regulation of *Synechocystis* ammonium permeases. In order to verify such an interaction, we performed pull-down assays using a C-terminal 3xFLAG-tagged P_II_ fusion protein that was expressed in *Synechocystis* under control of the strong *psbA* promoter. Protein extracts were prepared in presence of detergent, to solubilize membrane-bound proteins, and were subjected to anti-FLAG-tag immunoprecipitation. The proteome composition of the immunoprecipitate was subsequently analyzed by MS to reveal the proteins that co-purify with FLAG-tag P_II_. As an initial experiment, the immunoprecipitation was performed with cells grown in BG-11 medium, containing nitrate as unique nitrogen source. To discriminate between P_II_ interaction partners and unspecific background proteins, the non-transformed wild-type strain was used as an experimental control. Each experiment was further validated by an independent replicate.

**FIGURE 3:**
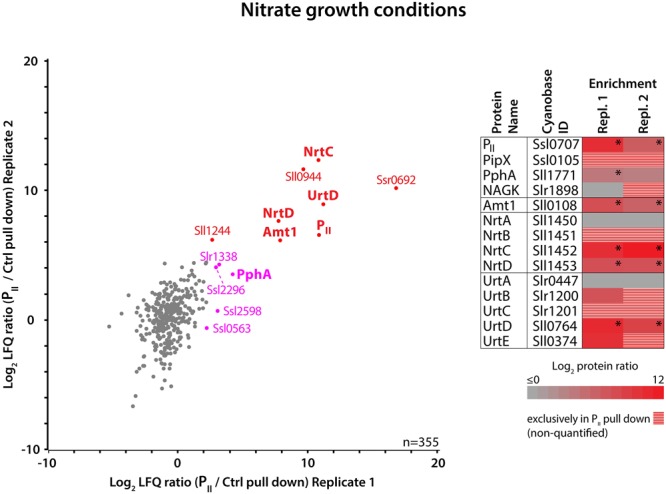
Proteins enriched in anti-FLAG-tag P_II_ immunoprecipitates from nitrate growth conditions. **(Left)** Scatterplot of the log_2_ transformed LFQ protein ratios (P_II_/control pull-down experiment) of 355 quantified proteins detected in two independent replicates. P_II_ pull-down specific outliers which are significantly enriched in both replicates are indicated in red, and significant outliers enriched only in Replicate 1 are indicated in magenta (*p*-value = 0.01). **(Right)** Heat map representation of the P_II_-specific enrichment of nitrogen metabolism related proteins. Log_2_ transformed LFQ protein ratios from both replicates are color coded and stars indicate significance. Proteins with missing LFQ ratios, detected exclusively in the P_II_ but not in the control pull-down, are striped in red/gray.

As a proof of concept, we were able to identify P_II_ as the most abundant protein in the P_II_ 3xFLAG pull-down (thereafter P_II_ pull-down). Furthermore, some reported interaction partners, such as PipX, were either exclusively identified in the P_II_ pull-down, or, as in the case of the P_II_ phosphatase PphA, were strongly enriched compared to the background control (Supplementary Figure 2) (Kloft and Forchhammer, 2005; Espinosa et al., 2007; Forcada-Nadal et al., 2014). The identification of a large number of background proteins in the experiments depends on the wash-stringency, the abundance of individual proteins and the high sensitivity of MS-detection. To distinguish between specific P_II_-enriched interaction partners and background, we determined relative protein abundance ratios between the P_II_ and control experiments by using label free quantification (LFQ) (Cox et al., 2014). Proteins with a significantly higher abundance in the P_II_ pull-down were identified by significance analysis (*p*-value = 0.01). Since both independent replicates from nitrate-grown cultures yielded very similar results, we correlated the data (Figure 3). Using this approach, the major ammonium permease Amt1 (Montesinos et al., 1998) indeed appeared among the significantly enriched proteins, supporting our suggestion that Amt1 represents a P_II_ binding target in cyanobacteria. Surprisingly, we could also identify subunits of other nitrogen import complexes besides Amt1 among the significantly enriched proteins, in particular subunits of the bispecific NrtABCD nitrate/nitrite and the UrtABCDE urea transporter complexes. For the NRT and URT transporters, all subunits except the periplasmic substrate binding proteins NrtA and UrtA were either exclusively present or strongly enriched in the P_II_ pull-downs. This suggests that the cytoplasmic components of the transporters together with the pore forming subunit are still associated in a complex, detached from the loosely associated periplasmic binding proteins (NrtA or UrtA).

**FIGURE 4:**
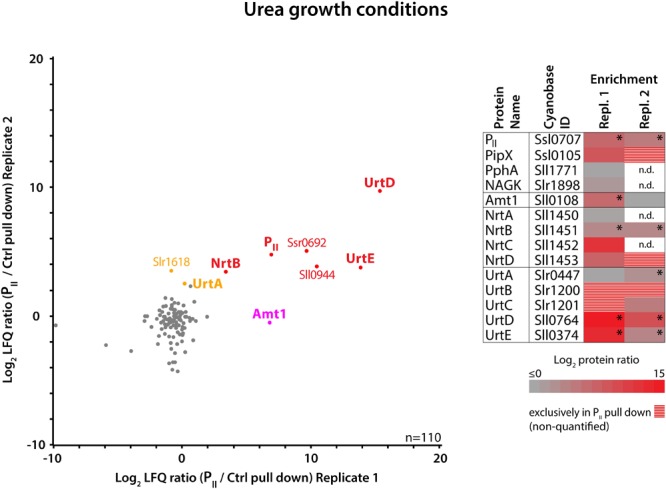
Proteins enriched in anti-FLAG-tag P_II_ immunoprecipitates from urea growth conditions. **(Left)** Scatterplot of the log_2_ transformed LFQ protein ratios (P_II_/control pull-down experiment) of 110 quantified proteins detected in two independent replicates. P_II_ pull-down specific outliers which are significantly enriched in both replicates are indicated in red, and significant outliers enriched in only one replicate are indicated in magenta (Replicate 1) or orange (Replicate 2) (*p*-value = 0.01). **(Right)** Heat map representation of the P_II_-specific enrichment of nitrogen metabolism related proteins. Log_2_ transformed LFQ protein ratios from both replicates are color coded and stars indicate significance. Proteins with missing LFQ ratios, detected exclusively in the P_II_ but not in the control pull-down, are striped in red/gray, and n.d. indicates no detection in P_II_ and control pull-down.

To further validate a potential interaction between P_II_ and the URT transporter complex, we repeated the experiment with cells grown in urea-supplemented medium. In agreement with the results from the P_II_ pull-down at nitrate growth conditions, we detected an enrichment of the P_II_ target PipX, the ammonium permease Amt1, subunits of the NRT nitrate/nitrite transporter, and most prominent, subunits of the URT urea transporter (Supplementary Figure 3). By correlating the data from both independent replicates (Figure 4), it became evident that UrtD and UrtE are the strongest enriched proteins under this condition besides P_II_. This suggests that the presence of urea in the medium influences the interaction of the URT transporter complex with P_II_.

Besides proteins associated with nitrogen import, we also identified two small hypothetical proteins to be significantly enriched in all experiments, Sll0944 and Ssr0692. Interestingly, both proteins are under transcriptional control of the global nitrogen regulator NtcA (Giner-Lamia et al., 2017), and reveal opposed dynamics in response to nitrogen availability. Under nitrogen-limiting conditions, Sll0944 was found to be strongly up-regulated at transcriptome and proteome levels, whereas Ssr0692 was strongly down-regulated (Spät et al., 2015; Giner-Lamia et al., 2017). Intriguingly, Ssr0692 seems to be up-regulated under carbon limitation (Battchikova et al., 2010) and might be associated to carbon fixation, as it was identified as a potential interactor of NdhH (Sato et al., 2007). The protein Sll0944 is highly conserved in cyanobacteria and possesses a domain of unknown function (DUF1830). Overall, our results imply a direct linkage of Sll0944 and Ssr0692 to nitrogen metabolism with a regulatory involvement of P_II_. Both proteins are currently under investigation to validate a potential interaction with P_II_ and to determine their function.

Surprisingly, the well-studied P_II_ interactor NAGK was not clearly enriched in these experiments. A low percentage of P_II_-NAGK complexes was expected to be present under nitrate or urea growth conditions (Burillo et al., 2004), although the strongest interaction occurs under conditions of nitrogen oversupply. Possibly, the C-terminal 3xFLAG-tag fusion of this P_II_ variant could negatively affect complex formation with NAGK. Therefore, pull-down experiments were also performed with an N-terminal 3xFLAG-tag fusion to P_II_. There, we could clearly identify NAGK in the pull-down of nitrate grown cells in two independent replicates (Table 4). The identification of NAGK in both N-terminal P_II_-fusion pull-down replicates implies that the localization of the 3xFLAG-tag has an influence on the interaction between P_II_ and its targets. Comparing the results from N- or C-terminal FLAG-tag fused P_II_ pull-down experiments can give additional information to identify potential P_II_ interaction partners. In contrast to the experiments utilizing the C-terminal FLAG-tag, where subunits of the heterotrimeric urease complex UreABC were not enriched, to our surprise, the N-terminal FLAG-tagged P_II_ protein co-purified the entire urease-complex including associated urease accessory proteins D, F, and G. This remarkable finding implies a possible direct interaction between the urease transport complex and the succeeding enzymatic machinery urea metabolism, indicating possible metabolic channeling of urea (Sweetlove and Fernie, 2018).

**Table 4:** Identified proteins in pull-down experiments at nitrate growth conditions utilizing the N-terminal 3xFLAG-tagged P_II_ fusion protein: Displayed are the identified proteins from two independent replicates (Repl. I and Repl. II).

Protein name/	Cyanobase	Mol. weight
complex	ID	(kDa)	iBAQ intensity	
			Repl. I	Repl. II
P_II_/N-3xFLAG P_II_	Ssl0707	15.43	4.2^∗^10^7^	1.1^∗^10^9^
PipX	Ssl0105	10.45	3.1^∗^10^6^	9.1^∗^10^7^
PphA	Sll1771	28.47	4.2^∗^10^4^	9.1^∗^10^5^
NAGK	Slr1898	31.53	9.4^∗^10^4^	6.5^∗^10^5^
Amt1	Sll0108	53.58	1.2^∗^10^8^	2.9^∗^10^8^
NrtA	Sll1450	48.97	1.1^∗^10^5^	1.4^∗^10^6^
NrtB	Sll1451	29.72	8.8^∗^10^5^	5.1^∗^10^6^
NrtC	Sll1452	75.10	1.4^∗^10^6^	6.1^∗^10^6^
NrtD	Sll1453	36.56	1.9^∗^10^5^	2.9^∗^10^6^
UrtA	Slr0447	48.36	1.0^∗^10^5^	2.6^∗^10^5^
UrtB	Slr1200	41.68	2.4^∗^10^5^	1.6^∗^10^6^
UrtC	Slr1201	45.08	3.5^∗^10^5^	4.9^∗^10^6^
UrtD	Sll0764	41.19	8.1^∗^10^5^	3.9^∗^10^6^
UrtE	Sll0374	27.42	6.5^∗^10^5^	6.4^∗^10^6^
UreA	Slr1256	11.06	–	4.9^∗^10^5^
UreB	Sll0420	11.38	2.0^∗^10^5^	5.2^∗^10^7^
UreC	Sll1750	61.04	9.9^∗^10^6^	3.5^∗^10^7^
UreD	Sll1639	27.16	1.2^∗^10^5^	6.4^∗^10^5^
UreF	Slr1899	20.23	3.1^∗^10^4^	5.2^∗^10^5^
UreG	Sll0643	22.01	1.3^∗^10^5^	1.2^∗^10^6^
Sll0944	Sll0944	18.15	8.4^∗^10^5^	9.8^∗^10^7^
Ssr0692	Ssr0692	5.85	2.8^∗^10^5^	9.3^∗^10^6^

*Protein intensities were calculated using the MaxQuant iBAQ algorithm (Schwanhausser et al., 2011).*

### Bacterial Two-Hybrid Assay

**FIGURE 5:**
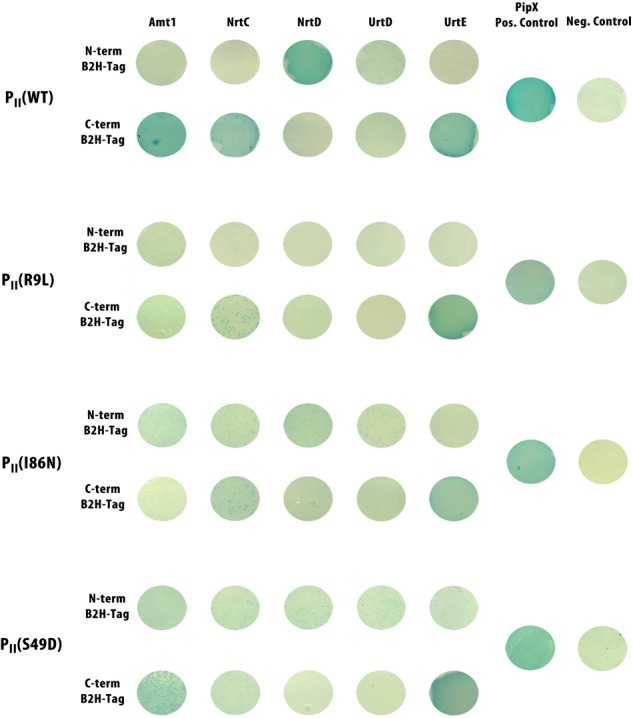
Bacterial-two-hybrid interactions on X-Gal plates of wild-type P_II_ and the P_II_ variants P_II_(R9L), P_II_(I86N), P_II_(S49D) with different transporter subunits. Interaction of P_II_ with C-terminal tagged PipX was used as a positive control. P_II_ with an empty pKT25 vector was used as negative control. Positive interactions are indicated by a blue coloration of the colonies.

To further confirm the interaction of P_II_ with the identified nitrogen transporters, we performed bacterial two-hybrid assays using the *Bordetella pertussis* adenylate cyclase two-hybrid system (BACTH) (Battesti and Bouveret, 2012). We tested the cytoplasmic localized ATP-binding proteins of the ABC-type transporters NrtC, NrtD, UrtD, UrtE (Omata et al., 1993; Valladares et al., 2002) as well as Amt1 for possible interactions with various P_II_ proteins. For each interaction candidate, an N- and a C-terminal fusion to the T25 fragment was constructed. In case of a positive interaction with a P_II_-T18 fusion, cAMP is formed within a cAMP deficient *E. coli* host cell. The interactions were tested by plate assays on X-Gal and MacConkey reporter plates for highest detection sensitivity. The P_II_ – PipX and leucine zipper interactions were used as positive controls. P_II_-T18 fusions with an empty pKT25 vector were used as negative controls. Next to wild-type P_II_-T18 fusion, we also included T18 fusions of the P_II_(I86N), P_II_(S49D), and P_II_(R9L) variants to find out how these different P_II_ variants, including a phosphomimetic T-loop variant and variants with different effector binding properties would differ in potential P_II_ interactions. Figure 5 shows the observed interactions on X-Gal plates in the bacterial two-hybrid assays.

All P_II_ variants showed interaction with the known P_II_ interaction partner PipX. Wild-type P_II_ showed clear interaction with Amt1 C-terminally fused to the T25 fragment, while the variants P_II_(I86N) and P_II_(R9L) did not interact. The loss of interaction of P_II_(I86N) is in agreement with our previous conclusions regarding the impaired ammonium utilization of *Synechocystis* sp. strain P_II_(I86N). The phosphomimetic variant P_II_(S49D) showed weak interaction with Amt1. In case of the nitrate transport ATP-binding subunits NrtC and NrtD, the BACTH assay revealed clear interaction of wild-type P_II_ with both subunits. Intriguingly, P_II_ interacted only with NrtC when it was fused at its C-terminus with T25, whereas in the case of NrtD, P_II_ interacted with the N-terminal fusion. In contrast to wild-type P_II_, the P_II_(R9L) variant was completely unable to interact with any of the Nrt-proteins, while weak interactions were observed with variants P_II_(S49D) and P_II_(I86N). With the ATP-binding subunits of the urea transporter, no interaction was observed with the UrtD protein, neither with wild-type P_II_ nor with any of the tested variants. By contrast, the C-terminal T25 fusion of UrtE showed clear interaction with wild-type and all P_II_ variants. The fact that positive interactions only occurred with selective combinations of P_II_ variants and N-or C-terminal T25 fusions of target proteins supports the conclusion that the observed interactions are indeed specific.

### P_II_ Deletion Mutant and P_II_(I86N) Mutant Excrete Nitrite

The pull-down and BACTH assays shown above clearly indicated interaction of wild-type P_II_ protein with components of the uptake systems for ammonium, nitrate, and urea. The interaction with Amt1 likely resembles the known interaction of the P_II_ protein GlnK from heterotrophic bacteria with AmtB (Conroy et al., 2007; Gruswitz et al., 2007) and perfectly explains the physiological response of *Synechococcus* PCC 7942 (hereafter *Synechococcus*) and *Synechocystis* P_II_ mutants toward ammonium. Interaction of P_II_ with components of the NrtABCD nitrate/nitrite transporter had in fact been suggested previously by several physiological studies, which documented altered nitrate utilization properties in various *Synechococcus* and *Synechocystis* P_II_ mutants (Kobayashi et al., 1997; Lee et al., 1998, 2000; Kloft and Forchhammer, 2005). A characteristic phenotype of a P_II_ deletion mutant grown in the presence of nitrate is the uncontrolled uptake of nitrate and subsequent excretion of nitrite into the medium (Kloft and Forchhammer, 2005). Reduction of nitrate requires two electrons whereas six electrons are needed for the reduction of nitrite to ammonium. Because of the lower electron demand for nitrate reduction, nitrite reduction becomes limiting when insufficient reductant is available. Therefore, when nitrate uptake is uncontrolled, nitrite accumulates and is excreted. We wondered if the *Synechocystis* P_II_(I86N) strain shows a similar nitrite excretion phenotype than the P_II_ deficient mutant when grown on nitrate and if P_II_-Venus complements the nitrite excretion phenotype of the P_II_-deficient mutant.

**FIGURE 6:**
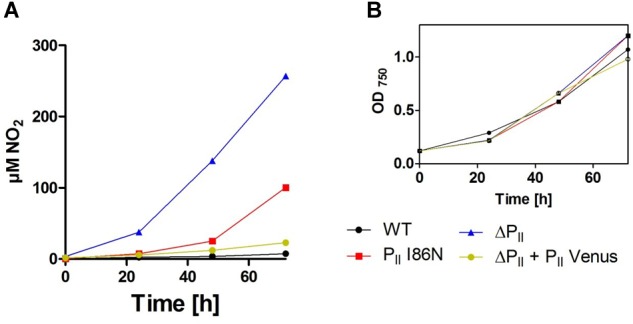
**(A)** Nitrite excretion of *Synechocystis* sp. wild-type, P_II_(I86N), ΔP_II_ and P_II_-Venus-comp. (ΔP_II_ + P_II_-Venus) complementation grown in BG11 medium supplemented with nitrate. **(B)** Corresponding growth curve to the experiment shown in panel **(A).**

To answer this question, *Synechocystis* wild-type strain, P_II_(I86N), ΔP_II_ and the P_II_-Venus comp. strains were grown with nitrate as sole nitrogen source under constant illumination (40 μmol photons m^-2^ s^-1^) and samples were removed for nitrite determination. Under these conditions, the various strains showed similar growth rates (Figure 6). Both mutant strains expected to be impaired in proper control of the NRT complex, the ΔP_II_ mutant and the P_II_(I86N) strain, indeed excreted nitrite into the medium. Notably, the P_II_(I86N) mutant excreted much lower nitrite amounts than the P_II_ deletion mutant, in agreement with the partial interaction of the P_II_(I86N) variant observed in the BACTH assays with NrtC and NrtD subunits. By contrast, P_II_-Venus perfectly complemented the nitrite excretion phenotype. Together, these results indicated that the BACTH interactions of P_II_ with the NrtC and NtcD subunits are physiologically meaningful.

### P_II_ Is Responsible for Inhibition of Nitrate Uptake

**FIGURE 7:**
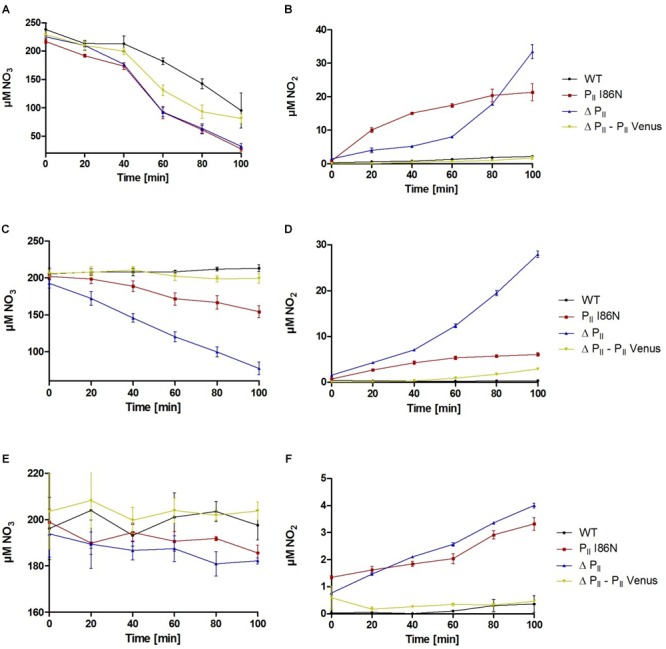
Nitrate utilization and nitrite excretion of various *Synechocystis* strains as indicated. Exponentially growing and nitrate supplemented *Synechocystis* cells were washed and resuspended in a medium containing 200 μM nitrate. Shown are the means of three biological replicates. **(A)** Nitrate utilization and **(B)** nitrite excretion under constant illumination of 40 μmol photons m^-2^ s^-1^. **(C)** Nitrate utilization and **(D)** nitrite excretion in presence of 2 mM ammonium and under constant illumination of 40 μmol photons m^-2^ s^-1^. **(E)** Nitrate utilization and **(F)** nitrite excretion in the dark.

To gain further insights in the P_II_-dependent regulation of nitrate uptake, we measured nitrate consumption rates of the various P_II_-mutants under different conditions. For this purpose, cells from exponential phase of growth in standard BG-11 medium were washed and subsequently incubated in BG-11^0^ medium containing 200 μM NO_3_ as nitrogen source. Nitrate utilization was quantified by measuring the nitrate concentration in the culture supernatant over time. Under constant illumination of 40 μmol photons m^-2^ s^-1^, *Synechocystis* wild-type and the P_II_-Venus comp. strain showed lower nitrate consumption rates, while both, the P_II_(I86N) and ΔP_II_ mutant, showed higher nitrate removal (Figure 7A). In agreement with our previous results, we observed excretion of nitrite by the P_II_(I86N) and ΔP_II_ mutants (Figure 7B). Interestingly, while the nitrate utilization rate of the P_II_(I86N) and ΔP_II_ mutant appeared almost identical, the two mutants differed in their rate of nitrite excretion. While nitrite excretion of the ΔP_II_ mutant accelerated over time, the P_II_(I86N) initially excreted nitrite faster but then slowed it down over time, explaining the lower amounts of nitrite in the exponentially growing culture (Figure 6) as compared to the ΔP_II_ mutant.

Addition of ammonium to nitrate grown cells leads to an immediate inhibition of nitrate uptake. This ammonium-dependent inhibition of nitrate uptake requires the presence of P_II_ protein or P_II_ phosphomimetic variants (Lee et al., 1998). Here, we tested if the P_II_(I86N) mutant was able to perform the ammonium-dependent nitrate uptake inhibition. For this purpose, nitrate consumption rates were determined after the addition of 2 mM ammonium. Both, *Synechocystis* wild-type and the P_II_-Venus comp. strains showed a complete inhibition of nitrate uptake in response to ammonium (Figure 7C). By contrast, the P_II_(I86N) and ΔP_II_ mutant strains maintained nitrate uptake after ammonium addition. However, nitrate uptake in the P_II_(I86N) mutant was clearly diminished as compared to the ΔP_II_ mutant (Figure 7C). The uncontrolled nitrate uptake of the P_II_(I86N) and ΔP_II_ mutant caused excretion of nitrite. Due to the diminished uptake of nitrate by the P_II_(I86N) mutant, its nitrite excretion was correspondingly lower as compared to the ΔP_II_ mutant (Figure 7D).

In addition to the absence of ammonium, active nitrate transport also requires photosynthetic CO_2_ fixation (Romero et al., 1985). Nitrogen assimilation is tightly regulated by light/dark transitions. A transition from light to dark causes an immediate inhibition of nitrate uptake and an inhibition of the ammonium assimilating GS (Romero et al., 1985; Marques et al., 1992). To reveal a role of P_II_ in this response, we tested the dark-switch-off of nitrate uptake in the various P_II_ mutant strains. Although the overall nitrate consumption was very low in darkness (Figure 7E) clear differences could be resolved. Notably, the P_II_(I86N) and ΔP_II_ mutant strains showed slow but constant nitrate uptake as compared to the wild-type and the P_II_-Venus comp. strain, which did not take up any nitrate. Corresponding to the uptake of nitrate, P_II_(I86N) and ΔP_II_ mutant excreted small amounts of nitrite (Figure 7F), supporting the notion that the P_II_(I86N) and ΔP_II_ mutants are unable to completely switch-off nitrate uptake in the dark.

### P_II_ Signaling Mutants Show Impaired Urea Utilization

**FIGURE 8:**
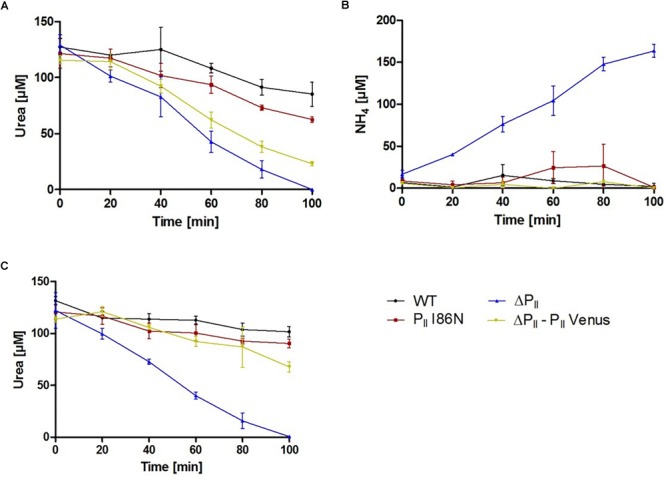
Urea utilization and ammonium excretion of various *Synechocystis* strains as indicated. Exponentially growing and nitrate supplemented *Synechocystis* cells were washed and resuspended in a medium containing 150 μM urea. Shown are the means of three biological replicates. **(A)** Urea utilization and **(B)** ammonium excretion under constant illumination of 40 μmol photons m^-2^ s^-1^. **(C)** Urea utilization in presence of 2 mM ammonium and under constant illumination of 40 μmol photons m^-2^ s^-1^.

To validate the physiological relevance of the interaction of P_II_ with the UrtE subunit of the urea transporter UrtABCDE, we analyzed urea utilization in the different P_II_ mutants. For this purpose, exponentially growing cells were washed and incubated in BG-11^0^ medium, containing 150 μM urea as sole nitrogen source with constant illumination of 40 μmol photons m^-2^ s^-1^. Under these experimental conditions, *Synechocystis* wild-type and P_II_(I86N) mutant strains utilized similar amounts of urea (Figure 8A), whereas both, the ΔP_II_ and P_II_-Venus comp. strains consumed considerably higher amounts of urea (Figure 8A). The wild-type property of the P_II_(I86N) variant is in agreement with the interaction of the P_II_(I86N) protein with the UrtE subunit, which appeared to be T-loop independent according to BACTH assays. Urea is hydrolyzed to CO_2_ and two molecules of ammonium by the enzyme urease (Mobley and Hausinger, 1989; Esteves-Ferreira et al., 2018). We wondered if an uncontrolled influx of urea could lead to an excretion of ammonium. Therefore, we measured ammonium concentrations in culture supernatant of those cells, which were incubated with 150 μM urea as the sole nitrogen source. Indeed, we detected ammonium excretion of the ΔP_II_ mutant proportional to its urea utilization (Figure 8B). In contrast, only minor amounts of ammonium could be detected in the culture supernatant of the *Synechocystis* wild-type, P_II_(I86N) and P_II_-Venus comp. strain (Figure 8B).

*Synechocystis* utilizes available nitrogen sources in a hierarchical order. In presence of ammonium, the uptake of external nitrogen sources is blocked (Muro-Pastor et al., 2005). To test ammonium-dependent inhibition of urea utilization, we determined urea consumption in presence of 2 mM ammonium. The *Synechocystis* wild-type, P_II_(I86N) and P_II_-Venus comp. strains showed a clear inhibition in urea utilization in response to the addition of ammonium. In contrast, urea uptake in the ΔP_II_ mutant was completely unaffected by ammonium addition (Figure 8A,C). This demonstrates that P_II_ also controls urea uptake, as suggested by the interaction assays.

### P_II_ Localization Changes Upon Addition of Nitrate, Ammonium, and Urea

Our previous pull-down experiments and bacterial two-hybrid assays clearly showed interaction of *Synechocystis* P_II_ with Amt1 as well as with nitrate and urea transport components. All interactions appeared to be physiologically relevant. Since all these transporters are localized to the plasma membrane, we suspected that this interaction might affect the cellular localization of P_II_. Therefore, we monitored P_II_ localization using the *Synechocystis* P_II_-Venus comp. strain grown under different nitrogen regimes.

**FIGURE 9:**
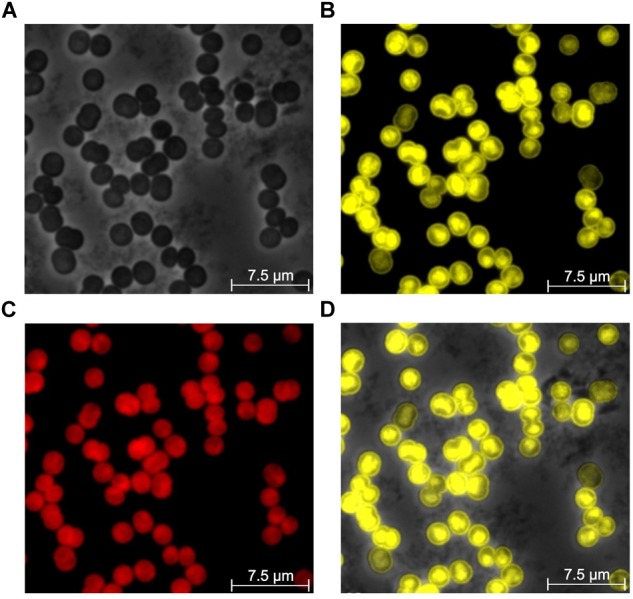
Localization of P_II_-Venus during exponential growth (OD_750_ of 0.5) of *Synechocystis* **(A)** Phase contrast image of cells. **(B)** Venus fluorescence. **(C)** Autofluorescence of thylakoid membranes. **(D)** Overlay of Venus fluorescence and phase contrast images.

Nitrate-replete cells in the mid-exponential growth phase (OD_750_ ∼ 0.5) showed heterogeneous distribution of P_II_-Venus fluorescence in the cell. The majority of cells displayed a strong fluorescence signal in their center and peripheral cell boundary (Figure 9A,B,D). The intense signal in the center corresponds to the central cytoplasmic space, inside the multiple thylakoid layers. In this region, P_II_ might interact with different soluble proteins, like NAGK or PipX. The area with low Venus fluorescence is occupied by the thylakoid membranes. The P_II_-Venus fluorescence at the cell boundary corresponds to the plasma membrane, where Amt1, NrtABCD and UrtABCDE are located (Hahn and Schleiff, 2014) (Figure 9C).

**FIGURE 10:**
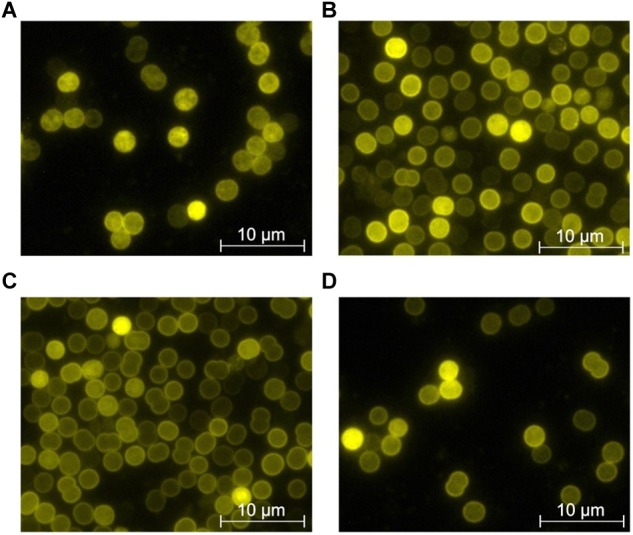
P_II_-Venus localization under different nitrogen supplemented conditions. **(A)** P_II_-Venus fluorescence of 1 week nitrogen starved *Synechocystis* cells. **(B–D)** P_II_-Venus fluorescence of *Synechocystis* during resuscitation from nitrogen starvation. One week nitrogen starved *Synechocystis* cultures were resuscitated by adding 5 mM nitrate **(B)**, 5 mM ammonium **(C)**, or 5 mM urea **(D)**. Fluorescence images were taken 1 h after addition of the nitrogen source.

When cells are shifted to nitrogen-depleted conditions, they activate the NtcA regulon, including the *glnB* gene and various uptake systems for nitrogen compounds. During prolonged starvation, they undergo chlorosis, which includes a reduction of thylakoid membranes (Forchhammer and Schwarz, 2018). The chlorotic cells rapidly respond to the addition of combined nitrogen sources. Therefore, investigation of P_II_-Venus fluorescence in chlorotic cells and following the addition of combined nitrogen sources was expected to reveal further insights in the *in vivo* dynamics of P_II_ interactions. To monitor the P_II_ localization in the chlorotic, nitrogen depleted state, cells were grown to an OD_750_ of 0.4–0.6, washed, and resuspended in BG-11^0^. After 1 week of nitrogen starvation, the P_II_-Venus signal was evenly distributed throughout the whole cell and its localization on the plasma membrane was not as distinct as during nitrate-supplemented exponential growth (Figure 10A). To test the localization following the addition of different nitrogen sources, 1 week nitrogen starved *Synechocystis* cultures were supplemented with 5 mM NO_3_, 5 mM NH_4_^+^ or 5 mM urea, respectively. Immediately thereafter, a change in the P_II_ localization became visible. The majority of cells showed a clearly visible, distinct plasma membrane localized P_II_-Venus fluorescence, while the remaining cytosol showed homogenously distributed fluorescence. The re-localization of P_II_ to the plasma membrane appeared most clearly in response to ammonium addition as compared to nitrate or urea (Figure 10B–D).

**FIGURE 11:**
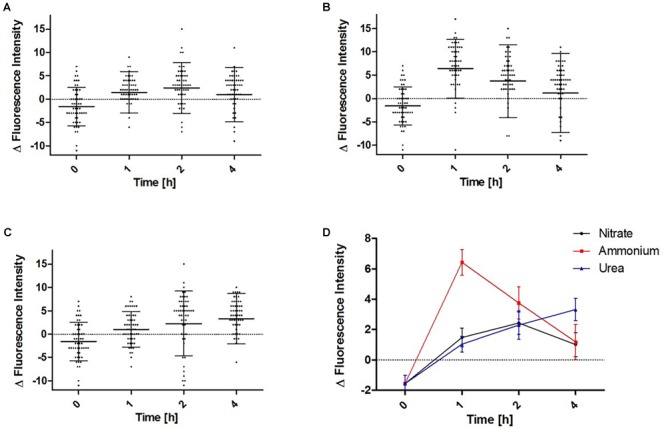
Quantification of the P_II_-Venus migration toward the plasma membrane in response to the addition of different nitrogen sources to nitrogen starved *Synechocystis* sp. cultures. A linear profile of the fluorescence intensities across midcell was recorded and evaluated as shown in Supplementary Figure 1. The maximum fluorescence intensity at the plasma membrane was normalized to the average intensity of the cytoplasm and then, the difference between plasma localized signal and average cytoplasmic signal was calculated (Δ fluorescence intensity). When Δ fluorescence intensity values are below 0, this indicates that the plasma membrane signal is weaker than the average cytoplasmic signal, while values above 0 indicate that the plasma membrane signal is higher than the average cytoplasmic signal. Resuscitation of 1 week nitrogen starved *Synechocystis* sp. cultures were induced by adding either 5 mM NO_3_ **(A)**, 5 mM NH_4_ **(B)**, or 5 mM urea **(C)**. 50–60 cells were measured per time point. Dots indicate single cell measurements; whiskers showing the standard-deviations; thick black lines show the arithmetic mean. **(D)** Direct comparison of the mean Δ fluorescence intensities from NO_3_, NH_4_, and urea induced resuscitation.

Since the fluorescence distribution is subject to a certain degree of heterogeneity with the bulk of single cells observed in the microscope, we attempted to quantitatively describe the average distribution of P_II_-Venus in a representative set of cells. Therefore, a profile of the fluorescence intensities across median cell sections was determined from 54 individual cells per investigated time point. From these profiles, the relative fluorescence intensity distribution between plasma membrane localized signal to the average cytoplasmic signal was determined and displayed as box-plot (Figure 11). Under nitrogen depletion, the majority of the cells showed a higher cytoplasmic P_II_-Venus localization than plasma membrane localization. Addition of a nitrogen source directly induced a re-localization of P_II_ toward the plasma membrane. In the first 2 h after addition of nitrate or urea, P_II_-Venus showed a similar localization change (Figure 11A,C,D). Two hours after the addition of nitrate, the P_II_-derived signal from the plasma membrane decreased again and re-appeared in the cytoplasmic space (Figure 11A), whereas in urea treated cells, P_II_ continued to accumulate at the plasma membrane (Figure 11C). Addition of ammonium induced the most prominent migration of P_II_-Venus to the plasma membrane in the first hour as compared to nitrate or urea, but thereafter, P_II_-Venus started to move back to the cytoplasm (Figure 11B).

## Discussion

The present investigation unraveled a so far unrecognized global function of the cyanobacterial P_II_ signaling protein in controlling the uptake of the major nitrogen sources ammonium, nitrate and urea. Previous studies already suggested occasionally involvement of P_II_ in the regulation of ammonium and nitrate transport (Kobayashi et al., 1997; Lee et al., 1998, 2000; Kloft and Forchhammer, 2005; Conroy et al., 2007), however, this issue was never addressed systematically using a combined biochemical, genetic and physiological approach. Starting from a series of pull-down experiments using FLAG-tagged P_II_ variants, we observed a putative interaction of P_II_ with various nitrogen transport systems. The interactions were verified by bacterial-two hybrid assays and the biological significance of the interaction was validated by physiological experiments. Finally, the proposed dynamic interaction of P_II_ with cytoplasmic membrane bound transporters was corroborated by fluorescence microscopy.

### Significance Statement

P_II_ signaling proteins play versatile roles in the coordination of carbon- and nitrogen anabolism in prokaryotes and plant chloroplast. In different phylogenetic lineages, P_II_ controls a variety of different target proteins. In cyanobacteria, the P_II_ paralog GlnB has been shown to control global nitrogen-responsive gene expression by interacting with the transcriptional co-activator PipX. Furthermore, P_II_ controls nitrogen storage metabolism in cyanobacteria by regulating the key enzyme of arginine synthesis, *N*-acetylglutamate kinase. Finally, a key enzyme in fatty acid metabolism, the acetyl-CoA carboxylase, was shown to be a target of P_II_. Several lines of evidences suggested that P_II_ might also be involved in the control of ammonium and nitrate uptake, however, direct involvement of P_II_ has not yet been shown. In this study, we revealed the interactome of P_II_ from the cyanobacterium *Synechocystis* by immunoprecipitating FLAG-tagged P_II_ protein. We found prominent enrichment of components of ammonium, nitrate and urea uptake systems. Direct protein–protein interaction was confirmed by bacterial-two hybrid analysis and the physiological relevance was verified by analyzing ammonium-, nitrate-, and urea-uptake in various P_II_ mutant strains of *Synechocystis.* This study, therefore, demonstrates that P_II_ is the master regulator of the most prominent nitrogen transport systems in cyanobacteria.

### *Synechocystis* P_II_ Regulates Ammonium Uptake by Interacting With the Amt1 Ammonium Permease

In *E. coli*, the P_II_ homolog GlnK regulates AmtB by direct protein–protein interaction to control the influx of ammonium (Conroy et al., 2007). In the GlnK-AmtB complex, the nucleotide binding pockets of GlnK are occupied with ADP and the T-loop of GlnK adopts a vertically extended conformation that closes the ammonium gas channel (Conroy et al., 2007; Maier et al., 2011; Forcada-Nadal et al., 2018). Since the conformation of the T-loop changes upon binding of ATP-Mg^2+^-2-oxoglutarate, the GlnK-AMT complex only forms under conditions of low 2-OG concentrations, which allows formation of the alternative GlnK-ADP complex (Radchenko et al., 2014).

The present results suggest that in *Synechocystis*, P_II_ regulates the major ammonium permease Amt1 in a similar manner than GlnK the AmtB channel. A weak interaction of Amt1 could still be detected with the phosphomimetic variant S49D. The reduced affinity indicates that the negative charge at position 49 reduces the affinity to Amt1. Therefore, S49 phosphorylated wild-type P_II_ is expected to have an even weaker affinity to Amt1 (two negative charges of phosphoserine as compared to one negative charge of aspartate). In this respect, phosphorylation of S49 would be analogous to uridylylation of Y51 in enterobacterial GlnK proteins, which prevents AmtB interaction (Conroy et al., 2007). Under conditions of strong P_II_ phosphorylation (nitrogen-poor conditions or high CO_2_ to nitrate ratio), the Amt1 channel would remain open, allowing unrestricted uptake of ammonium ions. When cells are shifted to excess ammonium conditions, P_II_ becomes dephosphorylated (Ruppert et al., 2002) and diminished 2-OG levels allow formation of the P_II_-ADP complex, which then can close the Amt1 pores.

In BACTH assays, the P_II_(R9L) variant was completely impaired in Amt1 interaction. This variant is also unable to interact with NAGK, presumably due to a stabilizing function of the R9 residue at the interface to the binding partner (Fokina et al., 2010a). The failure of this variant to interact with Amt1 indicates that also for Amt1 interaction, the lower face of P_II_ with the protruding T-loops is involved in this protein–protein interaction, in agreement with the structure of the GlnK-AmtB complex.

The failure of the P_II_(I86N) variant to interact with Amt1 corresponds to the loss of its affinity toward ADP (Fokina et al., 2010b). P_II_(I86N) has one order of magnitude higher affinity toward ATP than toward ADP and thus, is expected to reside almost exclusively in the ATP-bound state, which would abrogate the interaction with Amt1.

Despite that ammonium uptake in the *Synechocystis* P_II_(I86N) variant is no more under P_II_ control, this strain exhibits a higher ammonium tolerance than the ΔP_II_ mutant. This difference could be explained by different ammonium assimilation properties. In the P_II_ deletion mutant, the ammonium-scavenging arginine synthesis pathway is not active due to lacking activation of the key enzyme NAGK by P_II_ (Maheswaran et al., 2006). By contrast, *Synechocystis* P_II_(I86N) highly enhances NAGK activity, resulting in high intracellular arginine concentrations that leads to accumulation of cyanophycin granules (Watzer et al., 2015). This pathway will foster the metabolic removal of excess ammonium and thus results in increased ammonium tolerance.

Analysis of the subcellular localization of P_II_ in response to ammonium stimuli agrees with the above-depicted concept. Only 1 h after the addition of ammonium to chlorotic cells, P_II_ shows a strong migration toward the plasma membrane. Providing ammonium to nitrogen-starved cells should cause a strong decrease of the cellular 2-OG levels. This leads to the observed accumulation of P_II_ to the cytoplasmic membrane, preventing an excess uptake of too much ammonium. After a while, a new equilibrium will be established leading to a partial re-localization of P_II_ to its cytoplasmic targets, such as NAGK. Indeed, *Synechocystis* cells recovering from nitrogen chlorosis start to produce cyanophycin already after a few hours (Watzer and Forchhammer, 2018).

### P_II_ Regulates Nitrate Uptake by Interacting With NrtD and NrtC Subunits

Several previous studies documented an involvement of P_II_ in the regulation of nitrate/nitrite uptake. Our data provide novel insights regarding the mechanism of P_II_-mediated regulation of the NrtABCD transporter. It appears that the NrtABCD complex is directly regulated by interaction of P_II_ with the cytoplasmic ATPase subunits NrtC and NrtD, as indicated by the co-isolation of wild-type P_II_ with both of these proteins. The inability of the P_II_(I86N) complemented strain to regulate nitrate utilization as well as the reduced interaction of P_II_(I86N) with NrtC and NrtD subunits in BACTH assays could in principle be explained by the altered T-loop conformation or by the different ligand binding properties of this variant, discussed above. As shown by BACTH assays and by recent *in vitro* studies (Watzer and Forchhammer, unpublished), the P_II_(I86N) variant is indeed able to interact with PipX. This interaction requires a vertically extended conformation of the T-loop (Llacer et al., 2010; Forcada-Nadal et al., 2018). Although P_II_-PipX complexes are favored by the ADP-state of P_II_, efficient PipX-P_II_ interaction also occurs with the P_II_-ATP complex. As explained above, the P_II_(I86N) variant is trapped in the ATP-bound state. The fact that this P_II_ variant binds PipX indicates, that the T-loop of P_II_(I86N) is flexible enough to adopt various conformations. Therefore, it is likely that formation of the P_II_-NrtC or NrtD complex requires the ADP-bound state of P_II_, which is disabled in the P_II_(I86N) variant. As for NAGK and Amt1, the P_II_(R9L) variant is unable to interact with any of the NRT subunits, indicating that P_II_ interaction involves the lower part of the P_II_ body, where the T-loop emanates.

The phosphomimetic variant P_II_(S49D) shows, albeit weaker, interaction with the NRT subunits. This positive interaction explains previous reports, that *Synechocystis* and *Synechococcus* strains expressing these phosphomimetic variants, display a wild-type like regulation of nitrate/nitrite utilization (Lee et al., 2000; Kobayashi et al., 2005). Why the diminished interaction measured by BACTH assays does not properly reflect the physiological behavior of the P_II_(S49D) expressing strain (full complementation) could be due to different sensitivities of the assays. Kobayashi et al. (2005) used for the complementation of the *Synechocystis* deficient mutant plasmid-borne P_II_ constructs, which cause higher expression levels as in the wild-type genotype. Therefore, a weaker interaction of P_II_ with the NRT subunits could still be sufficient to inhibit NRT. The mechanism of NRT inhibition by P_II_ still awaits structure-functional explanation. Our BACTH assays indicate that P_II_ interacts with both ATPase subunits of NRT. Of these, the NrtC subunit seems to play a particular role: Truncation of the regulatory domain of NrtC results in an ammonium-insensitive NRT, despite the presence of a functional P_II_ system. The regulatory domain of NrtC possesses a putative binding site for nitrate (Koropatkin et al., 2006). Probably P_II_, through binding to NrtC and NrtD, must act in concert with the regulatory NrtC domain, to stop nitrate uptake. If one of the two regulators is missing, ammonium inhibition would not work. It is tempting to speculate that the regulatory domain of NrtC directly senses the nitrate-state of the cells (Koropatkin et al., 2006) to prepare NRT for inhibition by the P_II_-ADP complex, whereas P_II_ in the 2-OG-Mg-ATP complex (signaling high C/low N state) would not interact and, therefore, not inhibit NRT. This dual regulatory model combines information from the nitrate status with the global C/N and energy state sensing of P_II_ to tune NRT activity to the actual need. This model also explains the light-response of NRT: Low energy conditions favor the ADP-complex of P_II_, explaining why in the dark, complete switch-off of NRT requires P_II_.

### P_II_ Regulates Urea Uptake by Interacting With the UrtE Subunit

In addition to Amt1 and NRT, our data also identified the ABC-type urea transporter (UrtABCDE) as a novel P_II_ target, with the UrtE subunit being the direct interaction partner of P_II_. No BACTH interaction was observed for UrtD. This implies that the UrtD protein identified in the FLAG-tag pull-down resulted from pull-down of the entire URT complex. As deduced from the urea-utilization phenotype of the different P_II_ variant strains, as well as form the properties of BACTH interactions, the interaction of P_II_ with UrtE is distinct from the mode of Amt1 or NrtC and NrtD interaction. P_II_ interaction with UrtE seems to be T-loop independent, since the phosphomimetic S49D variant as well as the P_II_(I86N) and P_II_(R9L) variants interacted in BACTH assays like wild-type P_II_. The ability of P_II_(R9L) to interact with UrtE indicates that the interaction may involve a region of P_II_ distinct from the usual interaction face used by NAGK, PipX or Amt1.

In agreement with a different binding mode between P_II_ and UrtE, the P_II_(I86N) variant could substitute wild-type P_II_ regarding the control of urea utilization. This suggests that the P_II_-ATP complex is able to interact with UrtE in a non-conventional manner. The control of urea uptake prevents its futile hydrolysis and the consequent release of ammonium. Although the P_II_-Venus variant displayed higher urea uptake rates than the P_II_ deficient mutant, it could prevent concomitant ammonium excretion, indicating that also the P_II_-Venus variant successfully regulates urea utilization. Consistently, all the tested P_II_ variant strains (wild-type, I86N and P_II_-Venus) were able to carry out ammonium inhibition of urea uptake. Careful examination of the data shows that the P_II_-Venus strain displayed slightly higher urea uptake rates than the other two strains. It is possible that due to the bulky fluorescent proteins fused to P_II_ the interaction with P_II_ does not tune down urea uptake as efficient as in the case of the non-tagged P_II_ variants. Nevertheless, the P_II_-Venus construct is functional, which also agrees with the re-localization of P_II_-Venus to the plasma membrane upon addition of urea to nitrogen-starved cells. Under certain conditions, the entire multisubunit urease complex appeared in P_II_-FLAG-tag pull-down experiments. This observation deserves further investigation. It suggests either a direct interaction of the urease complex with P_II_ or a possible metabolic channeling between the urea uptake system and the multisubunit urease complex, leading to a co-immunoprecipitation of the entire super-complex. Further studies are required to reveal the molecular mechanism of urea metabolism and the involvement of P_II_ in this process.

## Conclusion

All together, the present study has expanded the insights in P_II_ regulatory interactions in *Synechocystis*. According to this study, the P_II_ regulatory network includes all relevant nitrogen uptake systems in *Synechocystis*. It is highly probable that the same holds true for other cyanobacteria. In *Synechococcus elongatus*, (Chang et al., 2013) genetic and physiological studies suggested that in addition to NRT, P_II_ is likely involved in the control of a cyanate ABC transporter (CynABC), which is not present in *Synechocystis*. Localization of P_II_-YFP fusions to the cytoplasmic membrane could also be seen in fluorescent images from *S. elongatus* (Espinosa et al., 2018). Control of transport proteins appears to be a highly conserved property of the P_II_ family members, as exemplified by the widely distributed GlnK-Amt interaction. Recently, a more distantly related member of the P_II_ superfamily, SbtB, an accessory component of the sodium-dependent bicarbonate transporter SbtA, was shown to be involved in regulation of bicarbonate metabolism in *Synechocystis* (Selim et al., 2018). Moreover, many putative non-characterized P_II_ family members are genetically linked to transport and channel proteins. However, except for GlnK-AmtB, no structures are known for P_II_-protein complexes with membrane channel proteins. Whether the interactions of the P_II_ members with the channels follow a universal mode, or whether different types of interaction exist, remains to be demonstrated. However, the differences observed between the interactions of P_II_ variants with UrtE, NrtC/D and Amt1 suggest that the interactions could be as versatile as observed for the various soluble P_II_ interactors (Forcada-Nadal et al., 2018).

## Data Availability

The datasets generated for this study can be found in the PRIDE ProteomeXchange consortium, PXD013411.

## Author Contributions

BW designed, conducted, and evaluated the cyanobacterial growth experiments, quantifications of nitrate, nitrite, ammonium, and urea utilization and carried out the microscopic experiments. RS constructed *Synechocystis* strains expressing tagged P_II_. PS and OH carried out anti-FLAG pull-down experiments. PS and BM carried out and evaluated nanoLC-MS/MS analyses. MK designed and constructed bacterial two-hybrid vectors. NN carried out and evaluated bacterial two-hybrid assays. KF designed and supervised the study and wrote the manuscript with BW. All authors gave input and approved the manuscript.

## Conflict of Interest Statement

The authors declare that the research was conducted in the absence of any commercial or financial relationships that could be construed as a potential conflict of interest.
