# Supplementary material for: The Signal Transduction Protein PII Controls Ammonium, Nitrate and Urea Uptake in Cyanobacteria
Source: Front Microbiol. 2019 Jun 25;10:1428. doi: 10.3389/fmicb.2019.01428 (PMC6603209; doi:10.3389/fmicb.2019.01428)
Supplement: Supplementary file 1 [file Presentation_1.pdf]

Supplementary Figures

The signal transduction protein P_{II} controls ammonium, nitrate and urea uptake in cyanobacteria

Björn Watzer [1§], Philipp Spät [1, 2§], Niels Neumann [1], Moritz Koch [1], Roman Sobotka [3], Boris Macek [2], Oliver Hennrich [1] and Karl Forchhammer [1*]

[1] Interfaculty Institute of Microbiology and Infection Medicine Tuebingen, Department of Organismic Interactions, Eberhard Karls Universität Tübingen, Tübingen 72076, Germany

[2] Interfaculty Institute for Cell Biology, Department of Quantitative Proteomics, Eberhard Karls Universität Tübingen, Tübingen 72076, Germany

[3] Centre Algatech, Institute of Microbiology, Academy of Sciences of the Czech Republic, Třeboň 379 01, Czech Republic

§ contributed equally

*** Correspondence:**
Karl Forchhammer
karl.forchhammer@uni-tuebingen.de

Supplementary Figure 1

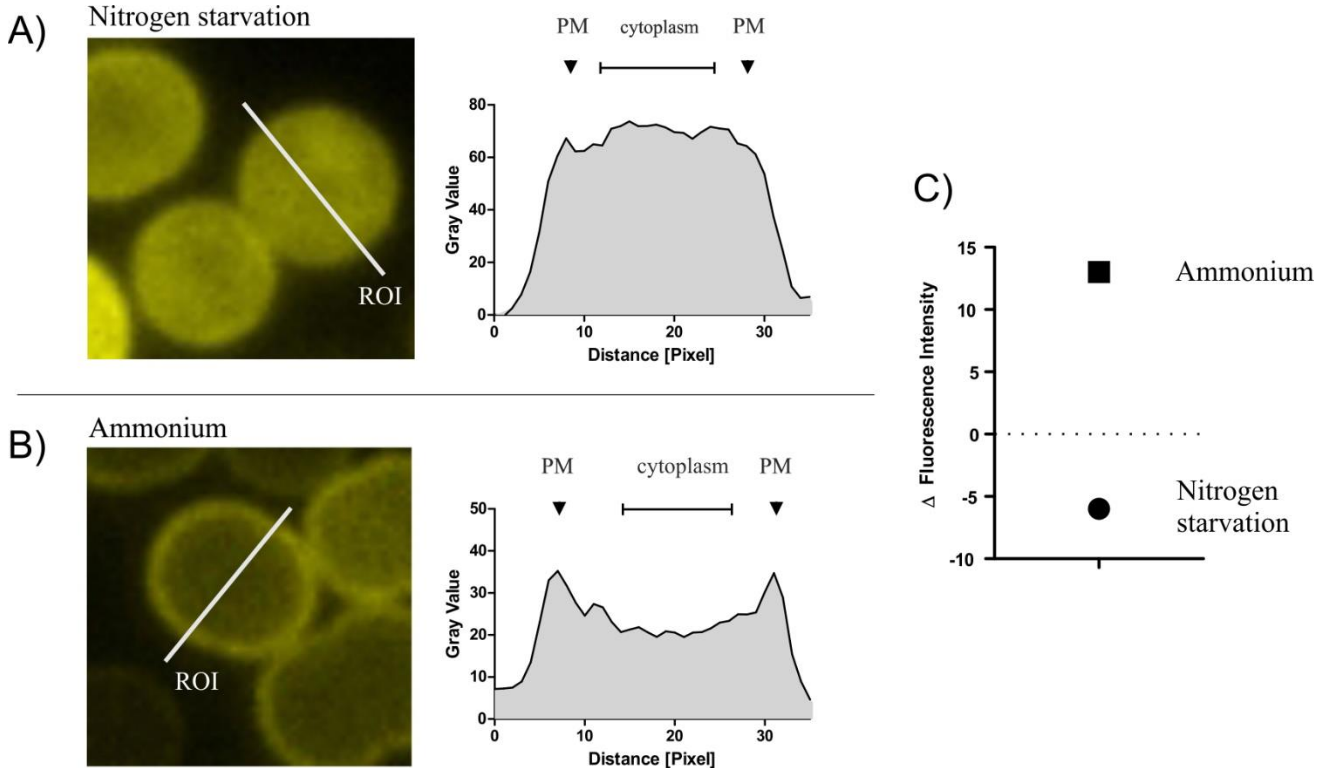

Supplementary Figure 1: Example of image evaluation of a nitrogen starved (A) and ammonium supplemented (B) *Synechocystis* cell. The fluorescents intensities of every pixel in a linear region of interest (ROI) across the cell were determined. The maximum gray value of the plasma membrane (PM) was subtracted from the average gray value of the cytoplasm (Δ Fluorescence intensity). (C) shows the final representation of the Δ Fluorescence intensity values. Values < 0 indicate a stronger cytoplasmic localized signal, while values > 0 show a stronger plasma membrane association.

Supplementary Figure 2

Nitrate growth conditions

Replicate 1

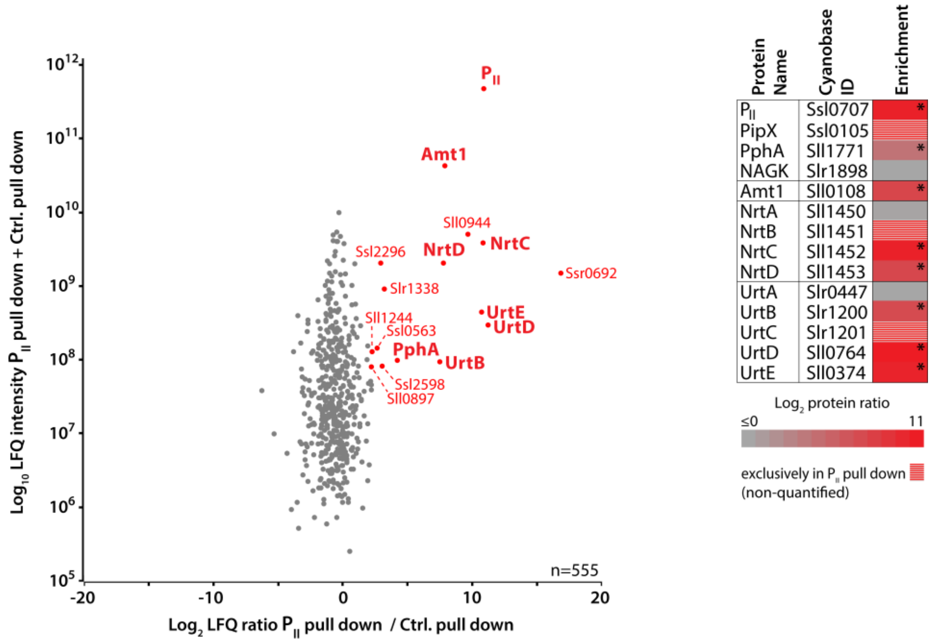

Replicate 2

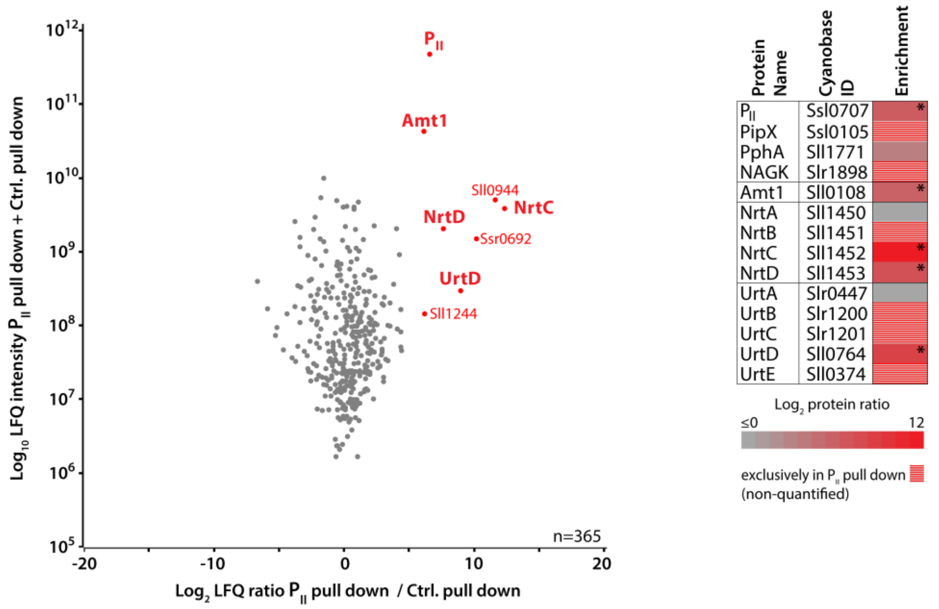

Supplementary Figure 2: Scatterplot of quantified proteins from two independent replicates at nitrate growth conditions (17.5 mM). Left: Log$_2$ transformed LFQ protein ratios (P$_{II}$/control pull down experiment) are plotted against the log$_{10}$ transformed sum of protein intensities. Significant outliers in P$_{II}$ pull down experiments (p value=0.01) are indicated in red. Right: Heat map representation of the P$_{II}$-specific enrichment of nitrogen metabolism related proteins. Log$_2$ transformed LFQ protein ratios are color coded with stars indicating significance. Proteins with missing LFQ ratios, detected exclusively in the P$_{II}$ but not in the control pull down, are displayed by red/grey stripes.

Supplementary Figure 3

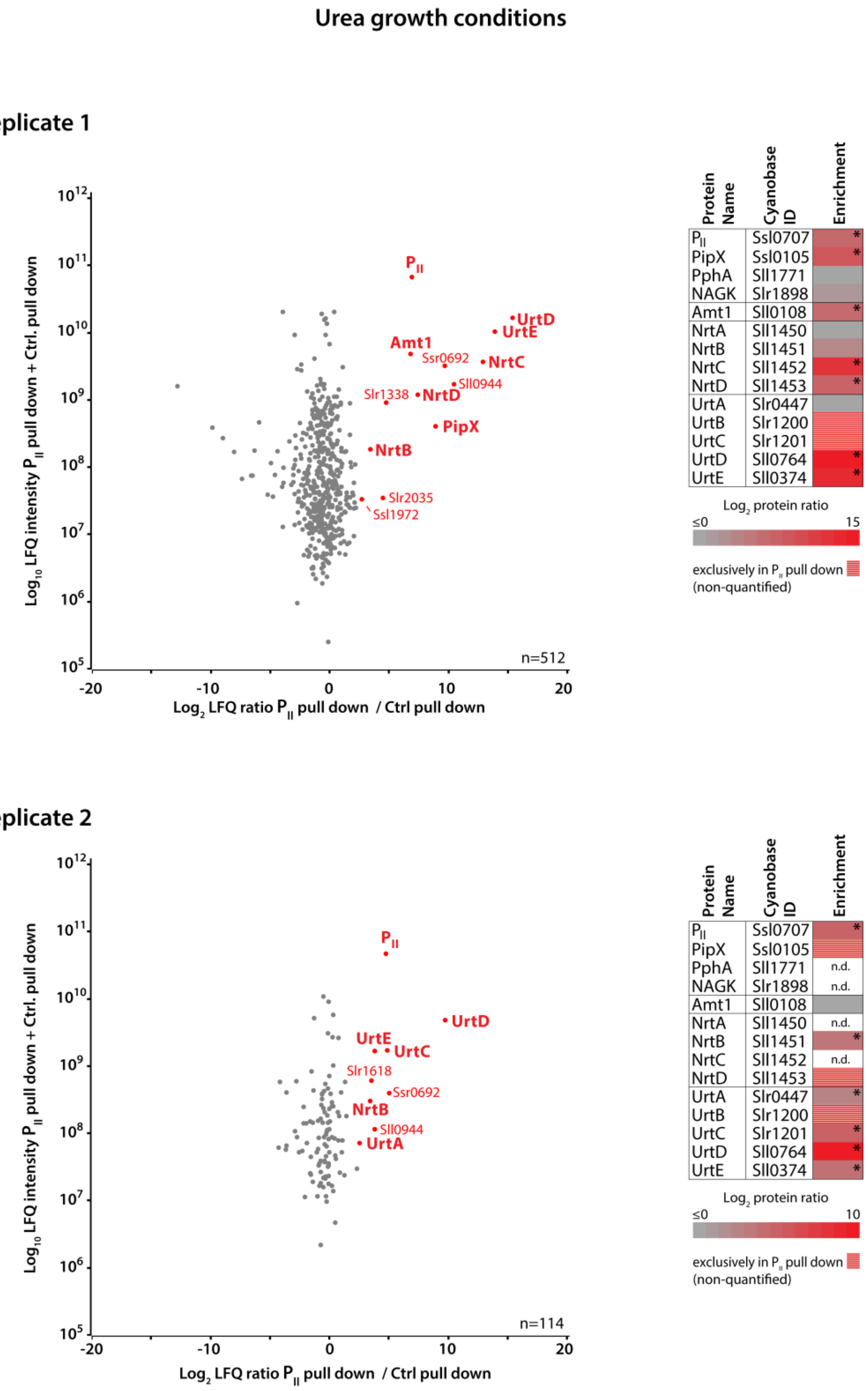

Supplementary Figure 3: Scatterplot of quantified proteins from two independent replicates at urea growth conditions (5 mM). Left: Log_2 transformed LFQ protein ratios (P_{II}/control pull down experiment) are plotted against the log_{10} transformed sum of protein intensities. Significant outliers in P_{II} pull down experiments (p value=0.01) are indicated in red. Right: Heat map representation of the P_{II}-specific enrichment of nitrogen metabolism related proteins. Log_2 transformed LFQ protein ratios are color coded with stars indicating significance. Proteins with missing LFQ ratios, detected exclusively in the P_{II} but not in the control pull down, are displayed by red/grey stripes, and n.d. indicates no detection in the P_{II} and control pull down.
